# An all-metallic nanovesicle for hydrogen oxidation

**DOI:** 10.1093/nsr/nwae153

**Published:** 2024-04-25

**Authors:** Juntao Zhang, Lujie Jin, Hao Sun, Xiaozhi Liu, Yujin Ji, Youyong Li, Wei Liu, Dong Su, Xuerui Liu, Zhongbin Zhuang, Zhiwei Hu, Qi Shao, Xiaoqing Huang

**Affiliations:** State Key Laboratory of Physical Chemistry of Solid Surfaces, College of Chemistry and Chemical Engineering, Xiamen University, Xiamen 361005, China; Innovation Laboratory for Sciences and Technologies of Energy Materials of Fujian Province (IKKEM), Xiamen 361005, China; Key Laboratory for Special Functional Materials of Ministry of Education, National & Local Joint Engineering Research Center for High-efficiency Display and Lighting Technology, School of Materials Science and Engineering, Collaborative Innovation Center of Nano Functional Materials and Applications, Henan University, Kaifeng 475004, China; Institute of Functional Nano and Soft Materials (FUNSOM), Jiangsu Key Laboratory for Carbon-Based Functional Materials & Devices, Soochow University, Suzhou 215123, China; State Key Laboratory of Rare Earth Resource Utilization, Changchun Institute of Applied Chemistry, Chinese Academy of Sciences, Changchun 130022, China; Beijing National Laboratory for Condensed Matter Physics, Institute of Physics, Chinese Academy of Sciences, Beijing 100190, China; Institute of Functional Nano and Soft Materials (FUNSOM), Jiangsu Key Laboratory for Carbon-Based Functional Materials & Devices, Soochow University, Suzhou 215123, China; Institute of Functional Nano and Soft Materials (FUNSOM), Jiangsu Key Laboratory for Carbon-Based Functional Materials & Devices, Soochow University, Suzhou 215123, China; State Key Laboratory of Rare Earth Resource Utilization, Changchun Institute of Applied Chemistry, Chinese Academy of Sciences, Changchun 130022, China; Beijing National Laboratory for Condensed Matter Physics, Institute of Physics, Chinese Academy of Sciences, Beijing 100190, China; State Key Lab of Organic-Inorganic Composites, Beijing University of Chemical Technology, Beijing 100029, China; State Key Lab of Organic-Inorganic Composites, Beijing University of Chemical Technology, Beijing 100029, China; Max Planck Institute for Chemical Physics of Solids, Dresden 01187, Germany; College of Chemistry and Chemical Engineering and Materials Science, Soochow University, Suzhou 215123, China; State Key Laboratory of Physical Chemistry of Solid Surfaces, College of Chemistry and Chemical Engineering, Xiamen University, Xiamen 361005, China; Innovation Laboratory for Sciences and Technologies of Energy Materials of Fujian Province (IKKEM), Xiamen 361005, China

**Keywords:** biomimetic synthesis, nanovesicle, interfacial strain, hydrogen oxidation reaction

## Abstract

Vesicle, a microscopic unit that encloses a volume with an ultrathin wall, is ubiquitous in biomaterials. However, it remains a huge challenge to create its inorganic metal-based artificial counterparts. Here, inspired by the formation of biological vesicles, we proposed a novel biomimetic strategy of curling the ultrathin nanosheets into nanovesicles, which was driven by the interfacial strain. Trapped by the interfacial strain between the initially formed substrate Rh layer and subsequently formed RhRu overlayer, the nanosheet begins to deform in order to release a certain amount of strain. Density functional theory (DFT) calculations reveal that the Ru atoms make the curling of nanosheets more favorable in thermodynamics applications. Owing to the unique vesicular structure, the RhRu nanovesicles/C displays excellent hydrogen oxidation reaction (HOR) activity and stability, which has been proven by both experiments and DFT calculations. Specifically, the HOR mass activity of RhRu nanovesicles/C are 7.52 A mg_(Rh+Ru)_^−1^ at an overpotential of 50 mV at the rotating disk electrode (RDE) level; this is 24.19 times that of commercial Pt/C (0.31 mA mg_Pt_^−1^). Moreover, the hydroxide exchange membrane fuel cell (HEMFC) with RhRu nanovesicles/C displays a peak power density of 1.62 W cm^−2^ in the H_2_-O_2_ condition, much better than that of commercial Pt/C (1.18 W cm^−2^). This work creates a new biomimetic strategy to synthesize inorganic nanomaterials, paving a pathway for designing catalytic reactors.

## Teaser

This work proposes a biomimetic synthesis of nanovesicles by utilizing the interfacial strain as the driving force to curl the ultrathin nanosheets into nanovesicles.

## INTRODUCTION

Ever since the discovery of the first cell, the fascinating superstructure of cells has attracted intense attention [1]. A vesicle, enclosing a volume within an ultrathin wall, plays a critical role in protecting the substance of DNA, regulation of growth and reproduction and cellular nutrient transport [2,3]. Similarly, the vesicle also plays an important role in natural organisms. For example, cabbage leaves locally shrink to generate a multilayer spherical structure, providing a powerful strategy for preventing energy loss in low temperature conditions [4]. In particular, the vesicular structure is also an ideal structural model for designing catalysts; the ultrathin wall can expose more active sites [5,6] leaving unsaturated coordination atoms exposed [7,8], and the microscopic sacs can encapsulate the reactive species [9–11]; all of these features are highly advantageous for catalytic applications. Nevertheless, although the vesicular structure is ubiquitous in biomaterials, artificial metal-based vesicles are seldom reported.

**Figure 1.**
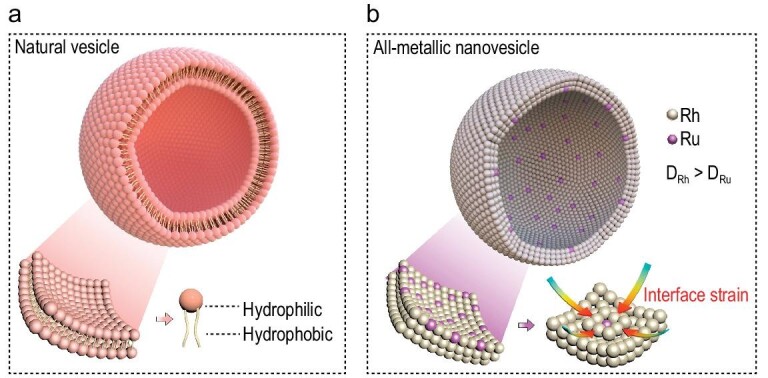
Formation mechanism diagram of (a) natural biological cell and (b) RhRu nanovesicles.

Nature is always the mentor of human beings. For centuries, the evolution of new structural materials has often been produced by imitating creatures living in the natural world [12–15]. For instance, Yu *et al.* have successfully fabricated bulk synthetic nacre by simulating the natural process of mollusks, which exhibits excellent ultimate strength and toughness [16]. Neinhuis *et al.* also found the superwettability system by studying the structure of lotus leaves [17]. Therefore, bio-inspired design of the cell structure is promising for constructing metal-based vesicle structural materials. In general, the cell structure is composed of an ultrathin bio-membrane, which owns an ultrathin structure and a dual hydrophilic-hydrophobic character [18]. The formation of bio-vesicles is highly controlled by the amphiphilic character of bio-membrane, in which one side is attracted to the polar situation and the other side is attracted to the non-polar situation [19], providing a delicate driving force for self-assembly into vesicles (Fig. 1a). Based on analysis of the formation of bio-vesicle structures, the bionic synthesis of metal-based vesicles is limited by some key questions including: (1) The synthesis of ultrathin metallic nanosheets is extremely difficult, mainly due to the fact that the anisotropic growth of metallic materials is thermodynamically unfavorable for the formation of ultrathin structures [20–22]. (2) Great lattice pressure must be overcome during the flexural growth process [23]. (3) Metallic nanosheets with a dual hydrophilic-hydrophobic character are almost nonexistent. While ultrathin metallic nanosheets can be obtained by wet-chemical strategies [24–27], the lack of a driving force for curling the metallic nanosheet is a key obstacle in the bionic synthesis of metallic nanovesicles.

To overcome these challenges, we proposed a biomimetic synthesis of nanovesicles by utilizing the interfacial strain as the driving force to curl the ultrathin nanosheets into nanovesicles (Fig. 1b). In detail, the relatively high standard reduction potential of Rh^3+^/Rh enables the Rh nanosheets to be reduced with the assistance of CO in the initial reaction stage, with subsequent formation of an RhRu overlayer on the surface of the substrate Rh layer, resulting in lattice mismatch at the interface between the substrate Rh layer and the RhRu overlayer. The generated interfacial strain gives a powerful driving force to curl RhRu nanosheets into nanovesicles. Additionally, the curling process inevitably leads to the generation of compressive strain in the exposed Rh surface. Benefiting from the unique vesicular structure and the lattice strain effect, RhRu nanovesicles/C can deliver a hydrogen oxidation reaction (HOR) mass activity of 7.52 A mg_(Rh+Ru)_^−1^ at an overpotential of 50 mV, which is 3.40, 39.4, and 24.19 times those of RhRu nanosheets/C (2.20 A mg_(Rh+Ru)_^−1^), Rh/C (0.19 A mg_Rh_^−1^) and commercial Pt/C (0.31 mA mg_Pt_^−1^). Moreover, RhRu nanovesicles/C only suffer 16% current density decay after continuous electrochemical testing for 20 000 s. Meanwhile, the RhRu nanovesicles/C-based membrane electrode assembly (MEA) displays a high peak power density (PPD) of 1.62 W cm^−2^ in the H_2_/O_2_ condition, much higher than that of commercial Pt/C (1.17 W cm^−2^).

## RESULTS AND DISCUSSION

### Morphological and structural characterizations

**Figure 2.**
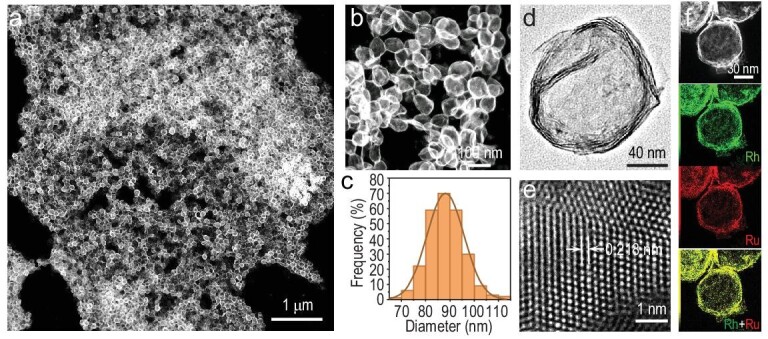
Morphology of RhRu nanovesicles. (a, b) HAADF-STEM images, (c) diameter distribution, (d) TEM image, (e) SA-HAADF-STEM image, and (f) SA-HAADF-STEM image with elemental mappings of RhRu nanovesicles (Rh in green and Ru in red).

RhRu nanovesicles were synthesized through a facile wet-chemical method, in which a homo-disperse solution of rhodium (III) acetylacetonate (Rh(acac)_3_), triruthenium dodecacarbonyl (Ru_3_(CO)_12_), potassium bromide (KBr), citric acid (CA), and oleylamine was heated at 160°C for 5 h (details in the Experimental Section). Transmission electron microscopy (TEM) and high-angle annular dark-field scanning TEM (HAADF-STEM) images revealed that the hollow nanovesicles were dispersed on the grid with a diameter of 89.5 nm (Fig. 2a-c and Fig. S1). High-magnification TEM imaging indicates that the nanovesicle is composed of several layers (Fig. 2d). The high sensitivity of nanovesicles to electron beam irradiation during the TEM tests indicated the superthin structure (Fig. S2). As revealed by the spherical aberration HAADF-STEM (SA-HAADF-STEM) image, the thickness of the single layer was measured to be 1.1 nm, being consistent with the high-magnification TEM results (Fig. S3). TEM energy-dispersive X-ray spectroscopy (TEM-EDS) revealed the ratio of Rh/Ru to be 95.5/4.5 (Fig. S4), which is highly similar to the inductively coupled plasma atomic emission spectroscopy (ICP-AES) result (95.0/5.0). According to the SA-HAADF-STEM image, the lattice space was measured to be 0.218 nm, which is slightly shorter than that of the (111) facet of Rh (Fig. 2e), indicating the existence of 1.5% compressive strain in RhRu nanovesicles. Corresponding fast Fourier transform (FFT) pattern reveals a face-centered cubic structure viewed from the [110] direction (Fig. S5). Simultaneously, HAADF-STEM-EDS mapping and line scan profiles suggest the homogenous distribution of Rh and Ru throughout the nanovesicle (Fig. 2f and Fig. S6).

**Figure 3.**
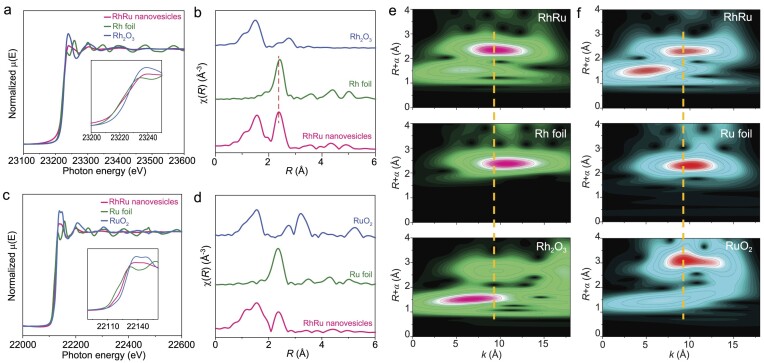
Structure of RhRu nanovesicles. (a) Rh K-edge XANES and (b) Rh K-edge FT-EXAFS of RhRu nanovesicles, Rh foil and Rh_2_O_3_. (c) Ru K-edge XANES and (d) Ru K-edge FT-EXAFS of RhRu nanovesicles, Rh foil, Ru foil and RuO_2_. (e) Rh K-edge WT-EXAFS spectra of RhRu nanovesicles, Rh foil and Rh_2_O_3_. (f) Ru K-edge WT-EXAFS spectra of RhRu nanovesicles, Ru foil and RuO_2_.

X-ray diffraction (XRD) pattern reveals that RhRu nanovesicles have a face-centred cubic structure, and the relative weak intensity of diffraction peaks further confirms the ultrathin structure (Fig. S7). Meanwhile, the diffraction peaks of RhRu nanovesicles upshift to a higher degree than those of the standard Rh crystals (PDF#05-2866), suggesting the existence of lattice compression. To further explore the structural and electronic properties of RhRu nanovesicles, X-ray photoelectron spectroscopy (XPS) and X-ray absorption spectroscopy (XAS) were carried out. The core-level Rh 3d XPS spectra show the coexistence of metallic and oxidized Rh in RhRu nanovesicles (Fig. S8A). The normalized Rh K-edge X-ray absorption near-edge structures (XANES) reveal that the absorption energy of RhRu nanovesicles is slightly higher than that of Rh foil (Fig. 3a); these results indeed confirm that Rh is slightly oxidized in RhRu nanovesicles, mainly due to the fact that the superthin structure is more readily oxidized under ambient conditions [28,29]. The Rh K-edge Fourier transforms of extended X-ray absorption fine structure (FT-EXAFS) reveals that RhRu nanovesicles display a Rh-Rh bond length of 2.37 Å, which is 0.05 Å shorter than that of Rh foil (2.42 Å), indicating about 1.6% compressive strain in RhRu nanovesicles. This result is consistent with the SA-HAADF-STEM result (Fig. 3b). The Ru K-edge XANES spectra reveal that the adsorption edges of Ru in RuRu nanovesicles are centered between the Ru foil and RuO_2_, indicating that the chemical state of Ru is partially oxidized (Fig. 3c), which is consistent with the core-level Ru 3p XPS results (Fig. S8B). The peak at 1.54 Å in EXAFS spectra of Ru can be assigned to the Ru–O coordination (Fig. 3d), while the other peak (2.37 Å) locates between those of Ru foil (2.34 Å) and Rh foil (2.42 Å), meaning that Ru atoms are atomically dispersed on the RhRu nanovesicles (Fig. S9). Additionally, the analysis of the wavelet transforms of EXAFS (WT-EXAFS) spectra, further confirmed this result (Fig. 3e and f). Meanwhile, EXAFS fitting results and specific coordination information are shown in Fig. S10 and Table S1. The scattering path of RhRu nanovesicles can be divided into Rh–O, Rh–Ru, Ru–O, and Ru–Rh. The coordination number of RhRu nanovesicles is much lower than those of metal and metallic oxide, mainly due to the superthin structure. The bond length of Rh–Ru in RhRu nanovesicles is shorter than that of Rh foil, suggesting the existence of compressive strain in RhRu nanovesicles. Moreover, the bond length of Ru–Rh in RhRu nanovesicles is between those of Rh foil and Ru foil, further confirming the mono-atomic dispersion of Ru in RhRu nanovesicles.

**Figure 4.**
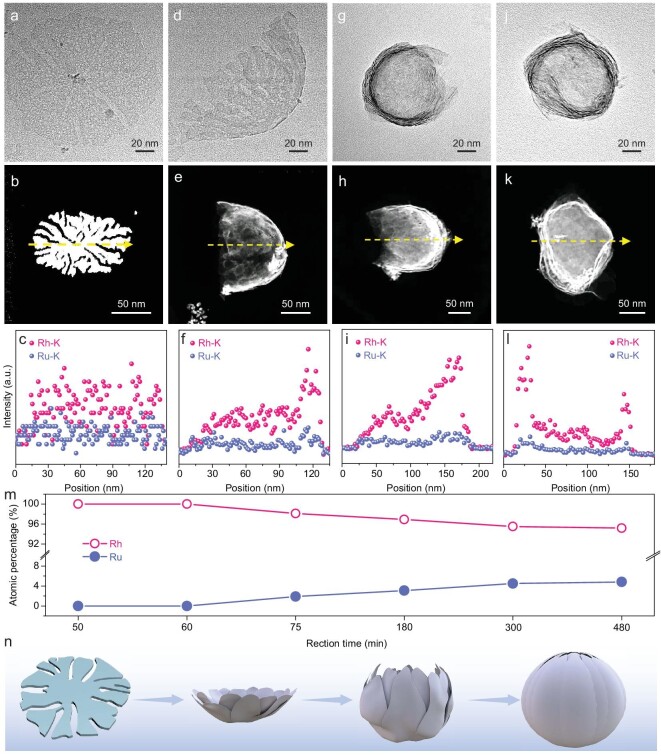
Growth mechanism and structure evolution of RhRu nanovesicles. (a, d, g, j) TEM images, (b, e, h, k) HAADF-STEM images, and (c, f, i, l) corresponding linear scanning analysis of the reaction intermediates collected at (a, b, c) 60 min, (d, e, f) 75 min, (g, h, i) 180 min, and (j, k, l) 300 min. (m) The ratio of Rh/Ru during the formation process. (n) The formation process of RhRu nanovesicles.

To study the formation mechanism of RhRu nanovesicles, morphologies and compositions of the reaction intermediates were initially collected by using TEM, HAADF-STEM-EDS line scan and TEM-EDS analysis, respectively. TEM image and corresponding HAADF-STEM-EDS reveal that only small snowflake-like NSs (with a diameter of below 20 nm) were formed after 50 min with no Ru element detected (Fig. S11), suggesting that the formation products were pure Rh at the beginning stage, mainly due to the fact that the standard reduction potential of Ru^3+^/Ru (0.45 V vs. reversible hydrogen electrode (RHE)) is much lower than that of Rh^3+^/Rh (0.76 V vs. RHE) [30–32]. As the reaction time goes on, the small nanosheets grow gradually along the lateral direction. The large number of nanosheets with a diameter of 150 nm lie flat on the TEM grid when the growth time reaches 60 min (Fig. 4a and Fig. S12A). The corresponding HAADF-STEM-EDS line scan profile and TEM-EDS indicate no Ru atom is reduced before this stage (Fig. 4b, c and Fig. S12B). Amazingly, the flat nanosheets begin to bend into a bowl-like structure as the reaction time increases to 75 min (Fig. 4d and Fig. S13A). As revealed by the HAADF-STEM-EDS line scan and TEM-EDS analyses, the Ru atom was slowly reduced at this stage (Rh/Ru = 98.1/1.9, Fig. 4e, f, and Fig. S13B). When prolonging the reaction time to 180 min, an unsealed vesicle is observed (Fig. 4g and Fig. S14A). Corresponding HAADF-STEM-EDS line scan and TEM-EDS analyses reveal that the ratio of Rh/Ru was 96.9/3.1 (Fig. 4h, i, and Fig. S14B). When the reaction time was further increased to 300 min, perfect multilayered nanovesicles with a Rh/Ru ratio of 95.5/4.5 were clearly observed (Fig. 4j-l and Fig. S15). The Ru content shows a slight increase when increasing the reaction time to 480 min (Rh/Ru = 95.2/4.8, Fig. S16). According to the HAADF-STEM-EDS line scan and TEM-EDS analyses in the formation of RhRu nanovesicles, we found that Ru elements undergo non-reduction, gradual reduction and reduction termination in the whole process (Fig. 4m). Based on the time-dependent structure and composition evolution analyses, the corresponding schematic drawing is shown in Fig. 4n, involving the initial formation of small pure-Rh nanosheets, growth of Rh nanosheets, and reduction of Ru atoms combined with the curling process.

To further discover the original reasons behind the curling process, a set of controlled experiments were carried out. In detail, only particulate nanostructures were obtained by removing Ru_3_(CO)_12_ or replacing Ru_3_(CO)_12_ with Ru(acac)_3_, whereas nanosheets were produced when Ru_3_(CO)_12_ was changed by HCHO, Fe_2_(CO)_9_, Co_2_(CO)_8_, Cr(CO)_6_, and Mo(CO)_6_ (Figs S17–S20). Meanwhile, CA also plays a critical role in producing the vesicular structure. Nanoparticles associated with NSs were obtained when we removed the use of CA (Fig. S21), and the quality of the nanovesicles decreased when replacing CA with ascorbic acid or glucose. Meanwhile, the reaction temperature, precursor concentration and the content of KBr also have a critical influence on the quality of the vesicular structure (Figs S22–S24). By analyzing the above experiments, we can draw the following conclusions: (1) CO, released from Ru_3_(CO)_12_, strongly controlled the growth of the 2D structures during the initial reaction process. (2) The introduction of Ru atoms plays a critical role in the curling growth of the nanosheet structure.

### Density functional theory (DFT) calculation

**Figure 5.**
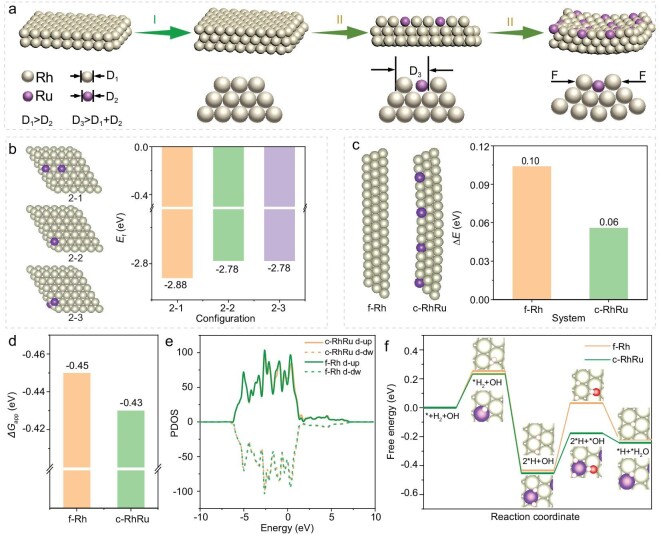
Curling mechanism of RhRu nanovesicles. (a) Schematic for the formation mechanism of RhRu nanovesicles. I and II represent the formation process of Rh nanosheet and RhRu nanovesicle, respectively. (b) Atomic models of different configurations of Ru atoms in the alloy slab and corresponding formation energies (*E*_f_) of different configurations from the pure Rh slab and reduced Ru atoms. (c) Geometric structure of the f-Rh model and the c-RhRu model and the electronic energy change (Δ*E*) after curling the model for the flat Rh/RhRu nanovesicle. (d) Δ*G*_app_ for c-RhRu and f-Rh. (e) PDOS of d-band of c-RhRu and f-Rh, where the Fermi level has been set to 0 eV; d-up/d-dw corresponds to the d electrons with spin up/down. (f) Free energy diagram of HOR pathway on f-Rh and c-RuRh.

Considering the nature of densest packing of noble metals [33], we used a rigid sphere to simulate Rh and Ru atoms. In order to illustrate the growth process intuitively, we performed the molecular model diagram to simulate the whole process. As shown in Fig. 5a, (I) in the early reaction stage, a free-standing pure Rh nanosheet was produced by the encapsulation effect of CO [34,35]. (II) As the reaction proceeds, reduced Ru atoms are deposited on the surface of substrate Rh nanosheets and alloyed with Rh atoms. To further explore the curling process, we carried out the DFT calculation. Fig. 5b shows the formation energy (*E*_f_) of the RhRu alloy slabs from the pure Rh slab and reduced Ru atoms. The configuration with 2 Ru atoms on the same side (Configuration 2–1) has lower *E*_f_ than those with 2 Ru atoms on different sides (Configurations 2–2, 2–3), confirming that the Ru atoms are more inclined toward ipsilateral growth. Since the fact that the Rh atomic radius (1.35 Å) is higher than that of Ru (1.30 Å) [36], the mutual attraction is much higher than the mutual repulsion between Ru and Rh atoms in the flat nanosheets, which induces the formation of interfacial strain between the RhRu overlayer and Rh substrate layer. Driven by interfacial stress, the nanosheets begin to bend in order to relieve the interfacial strain, leading to the compressive strain near the interface and tensile strain away from the interface in the substrate Rh layer, which is evidenced by the EXAFS analysis of RhRu nanovesicles. Meanwhile, we built multiple alloy slab models with different configurations of Ru atoms that substitute the Rh atoms on the same side, and calculated the *E*_f_ of the substitution defects (Fig. S25). Since the model with 7 Ru atoms (*N*_sd_ = 7) had the most negative formation energy, it was considered to have the optimal configuration and was selected for the subsequent building of the model for curling the RhRu nanosheet (see details in Materials and methods). Meanwhile, the curling of the RhRu nanosheet has been proven to be more favorable energy-wise than that of the Rh nanosheet (Fig. 5c), where the model of the curling RhRu nanosheet and that of the flat Rh nanosheet are abbreviated as c-RhRu and f-Rh, respectively. Herein, we used a simple model to describe the mechanism of the curling process (Fig. S26), where the dark red and light blue nanoplates represent RhRu and Rh nanosheets. From the side view, apart from the shear stress in the horizontal direction, the atomic interaction between Rh and Ru atoms also existed in the vertical direction. Both horizontal and vertical forces provide a powerful driving force to bend the nanosheet.

Meanwhile, DFT calculations were also carried out to predict the HOR performance on the c-RhRu and f-Rh. The Gibbs free energy changes (Δ*G*(H)/Δ*G*(OH)/Δ*G*(H_2_O), Fig. S27) are calculated for the binding between H/OH/H_2_O and c-RhRu/f-Rh, as they are common features for predicting the activity of HOR [37–39]. Although c-RhRu is similar to f-Rh in Δ*G*(H) and Δ*G*(H_2_O), c-RhRu has a Δ*G*(OH) (0.36 eV) that is obviously negative compared to that of f-Rh (0.46 eV), corresponding to an easier OH binding which is crucial for improving the activity of HOR [20]. Meanwhile, the descriptor, Δ*G*_app_ = Δ*G*(H) − Δ*G*(H_2_O) [20], for predicting HOR activity is also calculated. Compared with Δ*G*_app_ of f-Rh (−0.45 eV), that of c-RhRu (−0.43 eV) is slightly closer to the previously reported ideal value, i.e. Δ*G*_app_= 0 eV, which reflects the greater catalytic performance of c-RhRu (Fig. 5d). As shown in the projected density of states (PDOS, Fig. 5e), the d-band center (*E*_d_) of c-RhRu (−1.75 eV) is closer to the Fermi level in comparison with that of f-Rh (−1.78 eV), which should be caused by the Ru atoms and curling of the geometric structure. According to the d-band center theory and the previous research on HOR [40], the d-band with a higher energy center has a better orbital overlap with the p-orbital of the oxygen atom, which explains why the binding between oxygenous species and c-RhRu is stronger. Subsequently, according to the model proposed by Mao *et al*., the free energy diagram of HOR pathway on two kinds of substrates are calculated for a more direct comparison of kinetic performance [41]. This model includes 4 steps of elementary reactions, which are (1) the adsorption the of H_2_ molecule, (2) the dissociation of H_2_, (3) the adsorption of OH, and (4) the formation of free H_2_O molecules as shown in Fig. 5f. Steps (2) and (4) are exothermic, while steps (1) and (3) are endothermic. Step (3) is also the rate-determining step (RDS), since the highest energy barrier arises from this step. The energy barrier of c-RhRu (0.28 eV) is significantly lower than that of f-Rh (0.43 eV) in RDS, which theoretically indicates the superior activity of c-RhRu. These computational results suggest that the c-RhRu may have a greater HOR application potential than f-Rh.

### HOR performance in RDE and fuel cell

To this end, we performed HOR measurement of RhRu nanovesicles in an alkaline condition. To evaluate the electrochemical HOR performance, we deposited RhRu nanovesicles on the carbon support by sonication of RhRu nanovesicles and C solution (denoted as RhRu nanovesicles/C, Fig. S28) and benchmarked it against RhRu nanosheets/C (Fig. S29), home-made Rh/C and commercial Pt/C (Fig. S30). Figure S31 shows the cyclic voltammograms of these samples in N_2_-saturated 0.1 M KOH with a scan rate of 50 mV s^−1^. The peaks of underpotentially deposited hydrogen (H_upd_) for RhRu nanovesicles/C are lower than those of RhRu nanosheets/C, Rh/C and Pt/C, suggesting the weakest hydrogen binding energy (HBE) on RhRu nanovesicles/C. Meanwhile, the electrochemically active surface areas (ECSAs) of these samples, which were determined by CO stripping, also confirm the huge structural advantage of RhRu nanovesicles/C in electrocatalysis (Fig. S32). In detail, RhRu nanovesicles/C display an ECSA of 163 m^2^ g_(Rh+Ru)_^−1^, which is much higher than those of RhRu nanosheets/C (130 m^2^ g_(Rh+Ru)_^−1^), Rh/C (53 m^2^ g_Rh_^−1^) and Pt/C (63 m^2^ g_Pt_^−1^), suggesting the greater utilization of noble metals in RhRu nanovesicles/C. Based on the large ECSA of RhRu nanovesicles/C, we evaluated the electrocatalytic performance towards HOR. First, the HOR polarization curves of RhRu nanovesicles/C were conducted in H_2_ and N_2_-saturated 0.1 M KOH solution (Fig. S33). Anodic current over 0 V (RHE) is clearly observed in the H_2_-saturated electrolyte, while no anodic current is found in the N_2_-saturated electrolyte, suggesting the collected anodic current is mainly caused by H_2_ oxidation.

**Figure 6.**
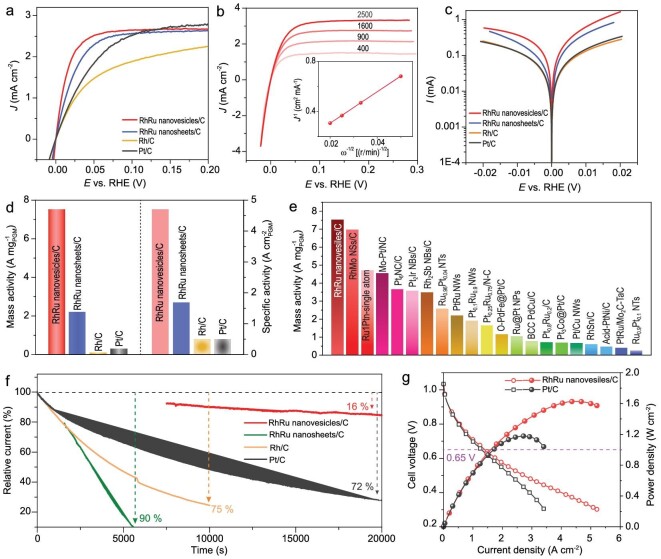
HOR performance. (a) HOR polarization curves of RhRu nanovesicles/C, RhRu nanosheets/C, Rh/C, and Pt/C in H_2_-saturated 0.1 M KOH. (b) HOR polarization curves of RhRu nanovesicles/C with various rotating speeds. Inset is the Koutecký–Levich plot of RhRu nanovesicles/C at an overpotential of 100 mV. (c) HOR Tafel plots and (d) mass activities and specific activities of RhRu nanovesicles/C, RhRu nanosheets/C, Rh/C, and Pt/C. (e) Comparison of the mass activity (at an overpotential of 50 mV) with the previous reports. (f) Relative current-time chronoamperometry responses of RhRu nanovesicles/C, RhRu nanosheets/C, Rh/C, and Pt/C in H_2_-saturated 0.1 M KOH at an overpotential of 100 mV vs. RHE. (g) Polarization and power density curves of H_2_-O_2_ HEMFCs with RhRu nanovesicles/C (0.4 mg_(Rh+Ru)_ cm^−2^) and commercial Pt/C (0.4 mg_Pt_ cm^−2^).

The HOR polarization curves of these samples were collected at a rotating speed of 1600 r/min with a scan rate of 5 mV s^−1^. As shown in Fig. 6a, RhRu nanovesicles/C displayed the highest anodic current densities among all the electrocatalysts under the whole potential range, suggesting the excellent HOR performance of RhRu nanovesicles/C. The HOR polarization curves collected with the rotation rate from 400 to 2500 r/min revealed that the limit current density increased with the rotation speed, mainly due to the enhanced H_2_ mass transport (Fig. 6b). Fitting by the Koutecký–Levich plots, the slope value for RhRu nanovesicles/C is 13.65 cm^2^ mA^−1^ (r/min)^1/2^, suggesting the anodic current is derived by the 2 e^−^ H_2_ oxidation (inset of Fig. 6b). The Tafel slope of kinetic current versus the overpotential were conducted. As shown in Fig. 6c, RhRu nanovesicles/C displayed the highest Tafel slope value among these samples, suggesting the highest kinetic process. Meanwhile, the mass activity for RhRu nanovesicles/C at an overpotential of 50 mV is 7.52 A mg_(Rh+Ru)_^−1^, which is much higher than those of RhRu nanosheets/C (2.20 A mg_(Rh+Ru)_^−1^), Rh/C (0.19 A mg_(Rh+Ru)_^−1^) and Pt/C (0.31 A mg_(Rh+Ru)_^−1^) (Fig. 6d). The HOR mass activity of RhRu nanovesicles/C is higher than most reported alkaline noble-metal–based HOR catalysts (Fig. 6e and Table S2). Meanwhile, RhRu nanovesicles/C also displays a specific activity of 4.71 mA cm^−2^, which is higher than those of RhRu nanosheets/C (1.69 mA cm^−2^), Rh/C (0.50 mA cm^−2^), and commercial Pt/C (0.49 mA cm^−2^). Moreover, electrochemical durability was evaluated by the long-time durability test, which was carried out by using the chronoamperometry measurement with a continuous operation at an overpotential of 100 mV. Significantly, RhRu nanovesicles/C maintains 84% of the initial current density after 20 000 s continuous test, which is higher than those of RhRu nanosheets/C (<10%), Rh/C (<25%) and Pt/C (28%) (Fig. 6f). Simultaneously, the initial morphology structure of RhRu nanovesicles/C was mainly preserved after 20 000 s *fig* analysis, while RhRu nanosheets/C, Rh/C and Pt/C were severely agglomerated (Fig. S34). Meanwhile, the crystal structure also remained after the durability test, indicating the excellent HOR durability of RhRu nanovesicles (Fig. S35).

Considering the high HOR mass activity of RhRu nanovesicles/C, the practical H_2_-O_2_ hydroxide exchange membrane fuel cell (HEMFC) performance was conducted. The MEA was acquired by employing the RhRu nanovesicles/C (0.4 mg_(Rh+Ru)_ cm^−2^) as the anode catalyst and the commercial Pt/C (0.4 mg_Pt_ cm^−2^) as the cathode, and the PAP-TP-85 as the membrane. For comparison, commercial Pt/C (0.4 mg_Pt_ cm^−2^) was employed as both the anode and cathode. Fig. 6g displays the polarization and PPD curves of RhRu nanovesicles/C and commercial Pt/C. The MEA based on RhRu nanovesicles/C displays a current density of 1.4 A cm^−2^ at 0.65 V (actual operating potential for automotive applications), which is higher than that of commercial Pt/C (1.2 mA cm^−2^) under H_2_-O_2_ condition. Meanwhile, the MEA catalyzed by RhRu nanovesicles/C also delivers a PPD of 1.60 W cm^−2^ than that of commercial Pt/C (1.18 W cm^−2^). The MEA based on RhRu nanovesicles/C also surpassed most previously reported HOR electrocatalysts (Table S3). Simultaneously, the MEA with RhRu nanovesicles/C exhibits a slight drop of current density throughout the 300 min test at a voltage of 0.7 V, suggesting a potential application of RhRu nanovesicles/C in HEMFC (Fig. S36).

## CONCLUSIONS

In summary, we demonstrated a unique biomimetic strategy for designing metallic vesicles, in which the interfacial strain played a powerful driving force to actuate the ultrathin nanosheets curling into nanovesicles. During the growth of the nanovesicles, the Rh nanosheet was initially formed, and the following reduced Ru atoms were alloyed with Rh depositing on the surface of the Rh nanosheet, resulting in the generation of interfacial strain between the RhRu overlayer and Rh substrate layer, which provided the driving force for nanosheets curling into nanovesicles. DFT calculation reveals that the surface Ru atom alloyed with Rh can drive the process. Meanwhile, the surface Ru alloy with Rh can reduce the energy barrier in HOR process. As a result, the RhRu nanovesicles/C display excellent HOR activity and stability, where the mass activity of RhRu nanovesicles/C at an overpotential of 50 mV is 7.50 A mg_(Rh+Ru)_^−1^, which is 3.40, 39.47, 24.19 times those of RhRu nanosheets/C (2.20 A mg_(Rh+Ru)_^−1^), Rh/C (0.19 A mg_(Rh+Ru)_^−1^), Pt/C (0.31 A mg_(Rh+Ru)_^−1^). Moreover, RhRu nanovesicles/C only displayed a slight current density decay of 16% even after 20 000 s of continuous operation, which is much lower than those of RhRu nanosheets/C (>90%), Rh/C (>75%), and Pt/C (72%), respectively. Moreover, the RhRu nanovesicles/C-based MEA displayed a high PPD of 1.62 W cm^−2^, possessing a potential application in HEMFC. This work shows that it is feasible to design new structures by a biomimetic strategy, which can draw rapid attention in biomimetic synthesis.

## METHODS

### Synthesis of RhRu nanovesicles

Ten mg Rh(acac)_3_, 50 mg Ru_3_(CO)_12_, 32 mg KBr, 100 mg CA and 5 mL oleylamine were mixed into a 30-mL glass vial. After ultrasonication for 1 h, the mixture was then heated from room temperature to 160°C and held at 160°C for 4 h in an oil bath. The products were collected by centrifugation and washed with a cyclohexane/ethanol (1/8) solution for several times.

### Synthesis of RhRu nanosheets

Ten mg Rh(acac)_3_, 120 mg PVP, 3 mL benzyl alcohol, and 3 mL formaldehyde were mixed into a 30-mL glass vial. After ultrasonication for 1 h, the mixture was transferred to a Teflon-lined stainless-steel autoclave and heated to 180°C and held at 180°C for 8 h. After cooling down to room temperature, 1.5 mg Ru(acac)_3_ was added into the autoclave and then heated to 180°C and held at this temperature for 4 h. The resulting black product was washed with a mixture of acetone and ethanol (8:1) for three times.

### Synthesis of Rh/C

Rh/C was synthesized by using the previously reported method [35]. Twenty mg RhCl_3_ and 50 mg carbon powder were dissolved in deionized water and ethanol and sonicated for 1 h. The mixture was dried by using a freeze dryer. Finally, the as-obtained powder was annealed at 600°C with a heating rate of 2°C/min in Ar atmosphere.

## Supplementary Material

nwae153_Supplemental_File
