# Supplementary material for: An all-metallic nanovesicle for hydrogen oxidation
Source: Natl Sci Rev. 2024 Apr 25;11(6):nwae153. doi: 10.1093/nsr/nwae153 (PMC11126156; doi:10.1093/nsr/nwae153)
Supplement: nwae153_Supplemental_File [file nwae153_supplemental_file.pdf]

Supporting information

An all-metallic nanovesicle for hydrogen oxidation

Juntao Zhang[1,2,3#], Lujie Jin[4#], Hao Sun[5#], Xiaozhi Liu[6#], Yujin Ji[4], Youyong Li[4], Wei Liu[5], Dong Su[6], Xuerui Liu[7], Zhongbin Zhuang[7], Zhiwei Hu[8], Qi Shao[9*], and Xiaoqing Huang[1,2*]

[1]State Key Laboratory of Physical Chemistry of Solid Surfaces, College of Chemistry and Chemical Engineering, Xiamen University, Xiamen, 361005, China.
Email: hxq006@xmu.edu.cn.

[2]Innovation Laboratory for Sciences and Technologies of Energy Materials of Fujian Province (IKKEM), Xiamen, 361005, China.

[3]Key Laboratory for Special Functional Materials of Ministry of Education, National & Local Joint Engineering Research Center for High-efficiency Display and Lighting Technology, School of Materials Science and Engineering, Collaborative Innovation Center of Nano Functional Materials and Applications, Henan University, Kaifeng, 475004, China.

[4]Institute of Functional Nano and Soft Materials (FUNSOM), Jiangsu Key Laboratory for Carbon-Based Functional Materials & Devices, Soochow University, Jiangsu, 215123, China.

[5]State Key Laboratory of Rare Earth Resource Utilization, Changchun Institute of Applied Chemistry, Chinese Academy of Sciences, Changchun, 130022, China.

[6]Beijing National Laboratory for Condensed Matter Physics, Institute of Physics, Chinese Academy of Sciences, Beijing, 100190, China.

[7]State Key Lab of Organic-Inorganic Composites, Beijing University of Chemical Technology, Beijing, 100029, China.

[8]Max Planck Institute for Chemical Physics of Solids, Nothnitzer Strasse 40, Dresden

01187, Germany.

[9]College of Chemistry and Chemical Engineering and Materials Science, Soochow University, Jiangsu, 215123, China.
Email: qshao@suda.edu.cn

[#]These authors contributed equally to this work.

Table of contents

METHODS

Figure S1. Low magnification TEM images of RhRu nanovesicles.

Figure S2. TEM images of RhRu nanovesicle after exposure to the electron beam for (a) 0 s and (b) 10 s.

Figure S3. (a) High magnification TEM and (b) SA-HAADF-STEM images of RhRu nanovesicles.

Figure S4. EDS spectrum of RhRu nanovesicles.

Figure S5. Corresponding FFT pattern of RhRu nanovesicles.

Figure S6. (a) HAADF-STEM image and (b) HAADF-STEM-EDS line scan profiles of RhRu nanovesicles.

Figure S7. XRD pattern of RhRu nanovesicles.

Figure S8. (a) Rh 3d and (b) Ru 3p XPS spectra of RhRu nanovesicles.

Figure S9. Enlarged Ru K-edge WT-EXAFS spectra of RhRu nanovesicles, Rh foil, and Ru foil.

Figure S10. FT-EXAFS fittings at R space of (a) Rh foil, (b) Rh_2O_3, (c) RhRu-Rh, (d) Ru foil, (e) RuO_2, and (f) RhRu-Ru.

Figure S11. (a) TEM image and (b) HTEM-EDS spectrum of RhRu nanovesicles intermediates obtained at the reaction time of 50 min.

Figure S12. (a) TEM image and (b) HTEM-EDS spectrum of RhRu nanovesicles intermediates obtained at the reaction time of 60 min.

Figure S13. (a) TEM image and (b) HTEM-EDS spectrum of RhRu nanovesicles intermediates obtained at the reaction time of 75 min.

Figure S14. (a) TEM image and (b) HTEM-EDS spectrum of RhRu nanovesicles intermediates obtained at the reaction time of 180 min.

Figure S15. (a) TEM image and (b) HTEM-EDS spectrum of RhRu nanovesicles intermediates obtained at the reaction time of 300 min.

Figure S16. (a) TEM image and (b) HTEM-EDS spectrum of RhRu nanovesicles intermediates obtained at the reaction time of 450 min.

Figure S17. (a, b) TEM images of products with the same reaction conditions as those of RhRu nanovesicles except the use of 0 mg $Ru_3(CO)_{12}$.

Figure S18. (a, b) TEM images of products with the same reaction conditions as those of RhRu nanovesicles except replacing $Ru_3(CO)_{12}$ with $Ru(acac)_3$.

Figure S19. TEM images of products with the same reaction conditions as those of RhRu nanovesicles except replacing $Ru_3(CO)_{12}$ with (a, b) $Fe_2(CO)_9$, (c, d) $Co_2(CO)_8$, (e, f) $W(CO)_6$, and (g, h) $Mo(CO)_6$.

Figure S20. (a, b) TEM images of products synthesized by using $Rh(acac)_3$ as the metallic precursor, HCHO as the reductant, and diphenyl ether and oleylamine as solvent.

Figure S21. (a, b) TEM images of the product by removing CA. TEM images of the products by replacing CA with (c, d) ascorbic acid and (e, f) glucose.

Figure S22. TEM images of the product synthesized with the reaction temperature of (a, b) 200 °C, and (c, d) 120 °C.

Figure S23. TEM images of the product synthesized by use of (a, b) 5 mg, and (c, d) 30 mg $Rh(acac)_2$.

Figure S24. TEM images of the product synthesized with (a, b) 0 mg , (c, d) 16 mg, and (e, f) 48

mg KBr.

Figure S25. Atomic models of different configurations of Ru atoms in the alloy slab and corresponding the formation energy (E_f) of substitution defects in different configurations.

Figure S26. Simplified model diagram of curling process from (A) side and (B) phantom view.

Figure S27. The Gibbs free energy (ΔG) changes before and after binding with H/OH/H_2O for f-Rh and c-RuRh.

Figure S28. (a, b) TEM images of RhRu nanovesicles/C.

Figure S29. (a, b) TEM images, and (c) HAADF-STEM image with EDS elemental mappings of RhRu nanosheets/C.

Figure S30. TEM images of (a) Pt/C and (b) Rh/C.

Figure S31. Polarization curves of RhRu nanovesicles/C, RhRu nanosheets/C, Rh/C, and Pt/C in N_2-saturated 0.1 M KOH at a scan rate of 50 mV s^{-1}.

Figure S32. CO stripping voltammograms of (a) RhRu nanovesicles/C, (b) RhRu nanosheets/C, (c) Rh/C, and (d) Pt/C in 0.1 M KOH at a scan rate of 10 mV s^{-1}.

Figure S33. Polarization curve of RhRu nanovesicles/C in N_2-satuated 0.1 M KOH.

Figure S34. TEM images of (a, b) RhRu nanovesicles/C, (c, d) RhRu nanosheets/C, (e, f) Rh/C, and (g, h) commercial Pt/C after 20000 s i-t tests.

Figure S35. H_2-O_2 HEMFC current density degradation vs. time of RhRu nanovesicles/C at the voltage of 0.7 V (RhRu nanovesicles/C in 0.4 $mg_{(Rh+Ru)}$ cm^{-2} in anode and commercial Pt/C (0.4 mg_{Pt} cm^{-2}, 20 wt% Pt/C) in cathode).

Table S1. Structure parameters extracted from the Rh K-edge EXAFS and Ru K-edge EXAFS fitting.

Table S2. Comparison of mass activity of RhRu nanovesicles/C with other previous reported PGM catalysts for HOR in 0.1 M KOH.

Table S3. Comparison of peak power density of HEMFC (H_2-O_2) operated with PGM-anode catalysts.

METHODS

Instrumentations. Transmission electron microscopy (TEM) was performed on JEOL (an accelerating voltage of 100 kV). High-angle annular dark-field scanning TEM (HAADF-STEM), HAADF-STEM energy dispersive X-ray spectroscopy (HAADF-STEM-EDS), and high-resolution TEM (HRTEM) were operated on FEI Tecnai F30 TEM at an accelerating voltage of 300 kV. Scanning electron microscope EDS (SEM-EDS) was conducted on ZEISS Sigma at an accelerating voltage of 20 kV. The inductively coupled plasma optical spectra (ICP-OES) was carried out on iCAP700 Thermo Fisher ICP-OES (Thermo Fisher Scientific, Waltham, MA USA) in axial mode. X-ray diffraction spectroscopy (XRD) was conducted on Rigaku with Cu Kα (λ = 1.540598 Å). X-ray photoelectron spectrum (XPS) was conducted on SSI S-Probe XPS spectrometer. XAS data were collected at ODE beamline of the Source Optimisée de Lumière à Energie Intermédiaire du LURE (SOLEIL, Grenoble, France), respectively. Data were processed according to standard procedures using the Demeter program package (Version 0.9.24).

Electrochemical Measurements. All the electrochemical measurements were performed at CHI760 electrochemical station (Chenhua, Shanghai) in a typical three-electrode system. A glassy carbon electrode (GCE) with diameter of 5 mm, graphite rod, and a saturated calomel electrode (SCE) were used as the working electrode, the counter electrode and the reference electrode, respectively. For the preparation of the catalyst ink, RhRu nanovesicles/C and Nafion solution (5 wt%) was mixed with isopropanol by ultrasonicated for 1 h. Then the homogenous ink was dropped on the GCE and the Rh and Ru loading was controlled as 3.0 $\mu g_{(Rh+Ru)}$ cm^{-2}, which is confirmed by ICP-OES. The equilibrium potential was the zero point of HER/HOR by using Pt/C as working electrode rotating at 1600 rpm in H_2-saturated electrolyte. All the polarization curves were corrected by solution resistance, which was tested by AC impedance spectroscopy from 200 kHz to 100 mHz. CO-stripping experiments were performed in 0.1 M KOH electrolyte. In detail, the CO adsorption was conducted by immersing the working electrode in 0.1 M KOH solution with bubbling CO (99.99%) gas for 20 min, then the electrolyte was bubbled N_2 for 15 min to remove the dissolved CO. Finally, two CV tests were conducted at a scan rate of 10 mV s^{-1}. The chronoamperometric measurements were conducted in H_2-saturated 0.1 M KOH at an overpotential of 0.1 V for 20000 s.

MEA test. Catalyst ink was prepared by mixing catalyst with 5 wt% PAP-TP-100 ionomers diluted by isopropanol/water (25:1) solvent. The catalyst ink was ultrasonic treated for 3 hour and sprayed on two side of PAP-TP-100 membrane (25 μm) to observed a catalyst-coated membrane (CCM) with an effective area of 5 cm^2 using airbrush. The RhRu nanovesicles/C was used as anode with a metal loading of 0.4 mg cm^{-2}, while the commercial 20 wt% Pt/C (Alfa Aesar, Johnson Matthey HiSpec 4000, 20 wt% Pt on Vulkan XC-72) was used as cathode with a loading of 0.4 mg_{Pt} cm^{-2}. The CCM and gas diffusion layer were assembled in a standard test cell of fixture

(2.25×2.25 cm^2). Then we tested them using Scribner 850e as condition-controlled fuel cell test station. Throughout the MEA tests, the fuel cell polarization curve was recorded at 95 ℃. While for fuel supply and oxygen supply, the back pressures were set to 200 kPa and gas flow rates were set to 500 mL min^{-1}.

DFT calculation. Based on the Rh (111) facet, we first constructed three-atomic-layered slab models for Rh and RhRu alloy systems, and the RhRu slabs are built by substituting several top-most atoms of Rh slab with Ru atoms in different configurations. Only the top-most layer atoms are allowed to relax in optimizing all the slab structures. To avoid interactions between periodic images, a vacuum layer of 15 Å is added to all the slab models. Then, the formation energy of the substitution defects (E_f(RhRu_slab)) is calculated for each possible RhRu slab model, and it is defined as;

E_f(RhRu_slab) = E(RhRu_slab)+N_{sd}*E(Rh_SA)-E(Rh_slab)-N_{sd}*E(Ru_SA),

where E(RhRu_slab), E(Rh_slab), E(Rh_SA), and E(Ru_SA) correspond to the electronic energies of RhRu slab, Rh slab, isolated Rh atom, and isolated Ru atom, respectively, while N_{sd} represents the number of the substitution defects. After confirming the energy-optimal RhRu slab by the formation energies, the flat RhRu NV is modeled by cutting the optimal RhRu slab into a nanoribbon, and the model of curling RhRu NV is built by curling the flat nanoribbon model directly. The modeling of flat/curling Rh NV also follows the similar way. We calculated the adsorption free energies (ΔG) on the nanoribbon models for H/OH/H_2O. ΔG is defined as $\Delta G = G(\text{total}) - G(\text{sub}) - G(\text{ad})$, where G(total) and G(sub) are the free energies of the nanoribbon model with and without the adsorbate, respectively, and G(ad) is the free energy of an adsorbate molecule. The free energy (G) can be obtained by the following formula: $G = E + E_{ZPE} - T\Delta S$, where E, E_{ZPE} and S represents the electronic energy, zero-point energy, and entropy of a system. T is the temperature. Our DFT calculations were carried out using the Vienna ab initio simulation package[42-44] and VASPKIT[45]. The PBE (Perdew-Burke-Ernzerhof) exchange-correction functional[46] was utilized in our calculations, and an energy cutoff of 400 eV was used for the plane-wave basis set. The Brillouin zone was sampled with the Γ-only scheme, and the convergence criterion for self-consistent optimization and atomic force minimum was 10^{-4} eV and 0.03 eV $Å^{-1}$, respectively.

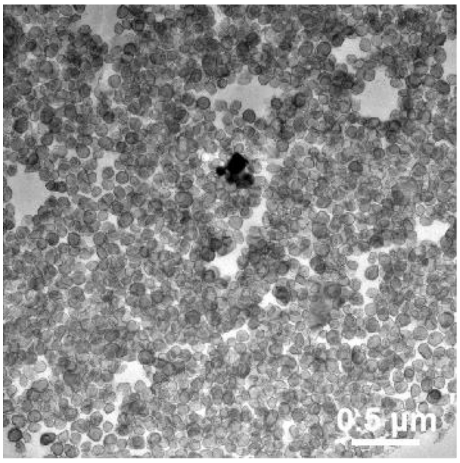

Figure S1. Low magnification TEM images of RhRu nanovesicles.

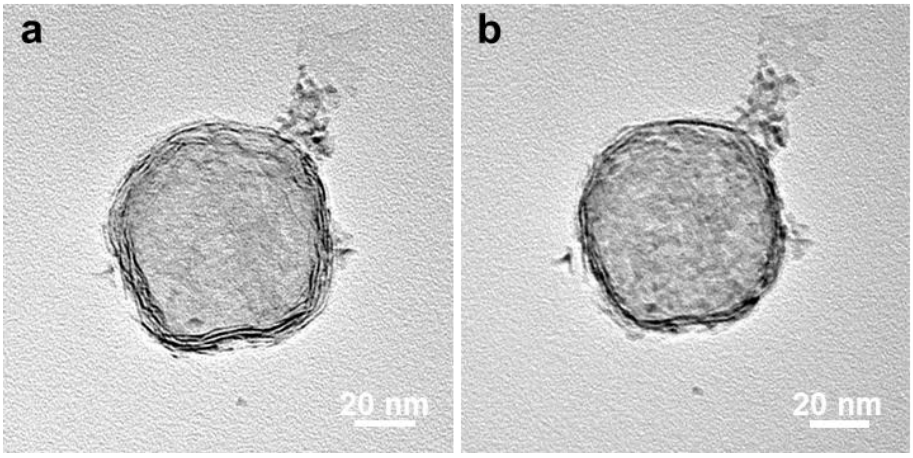

Figure S2. TEM images of RhRu nanovesicle after exposure to the electron beam for (a) 0 s and (b) 10 s.

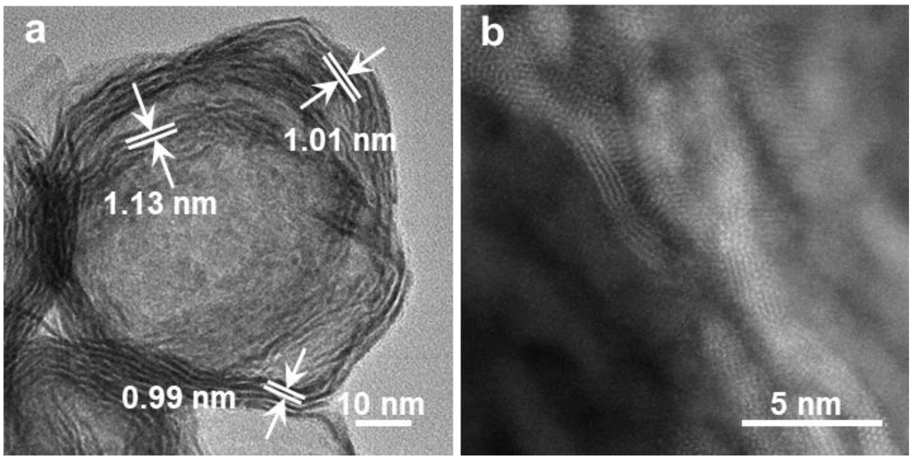

Figure S3. (a) High magnification TEM and (b) SA-HAADF-STEM image of RhRu nanovesicles. The thickness of single layer of RhRu nanvoesicles is around 1.1 nm.

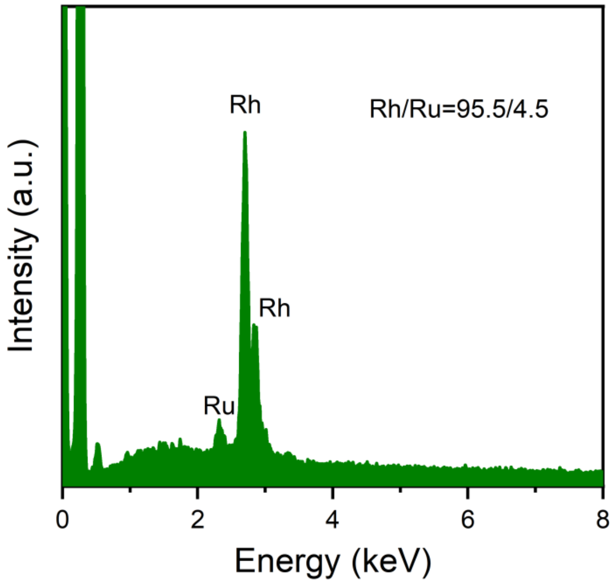

Figure S4. EDS spectrum of RhRu nanovesicles.

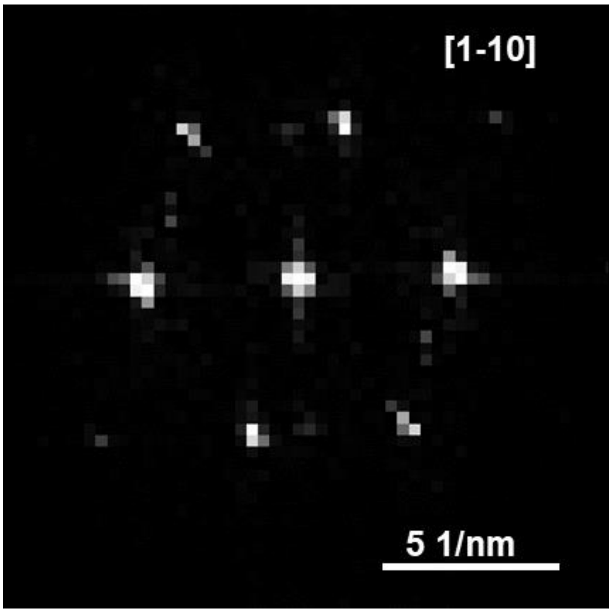

Figure S5. Corresponding FFT pattern of RhRu nanovesicles.

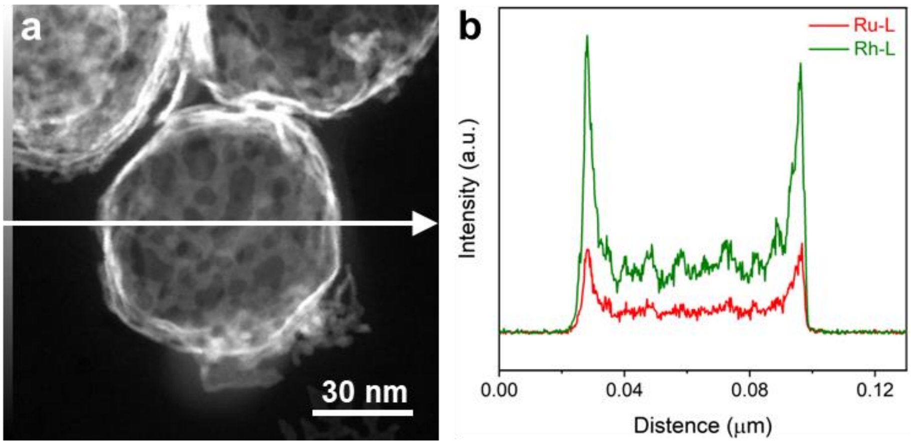

Figure S6. (a) HAADF-STEM image and (b) HAADF-STEM-EDS line scan profiles of RhRu nanovesicles.

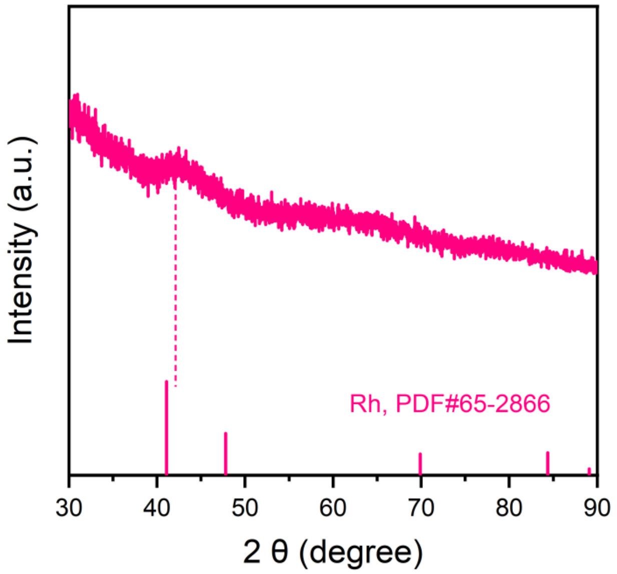

Figure S7. XRD pattern of RhRu nanovesicles.

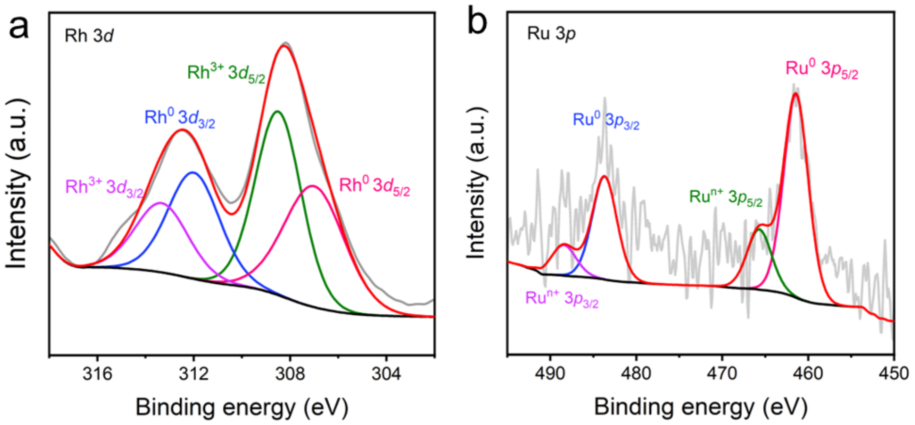

Figure S8. (a) Rh 3d and (b) Ru 3p XPS spectra of RhRu nanovesicles.

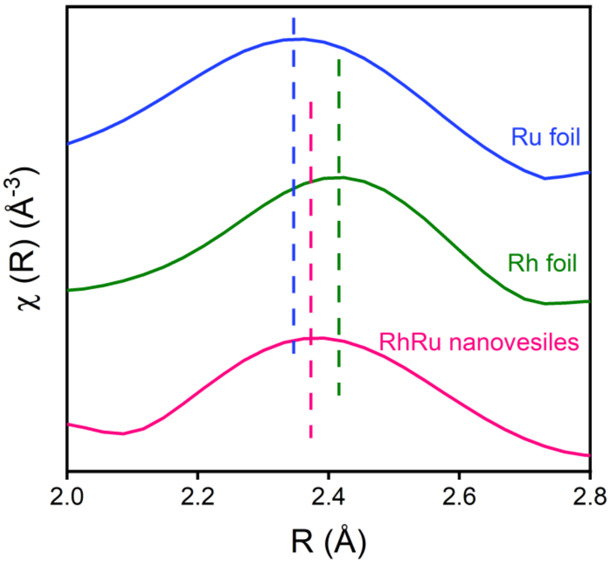

Figure S9. Enlarged Ru K-edge WT-EXAFS spectra of RhRu nanovesicles, Rh foil, and Ru foil. These result reveals that the position of Ru-Rh for RhRu nanovesicles locates between those of Ru foil and Rh foil, suggesting the atomic dispersed Ru in RhRu nanovesicles.

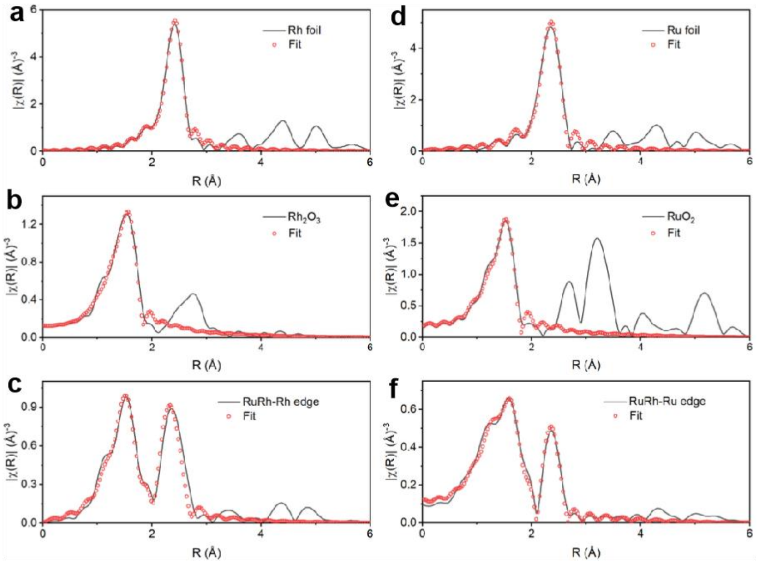

Figure S10. FT-EXAFS fittings at R space of (a) Rh foil, (b) Rh_2O_3, (c) RhRu-Rh, (d) Ru foil, (e) RuO_2, and (f) RhRu-Ru.

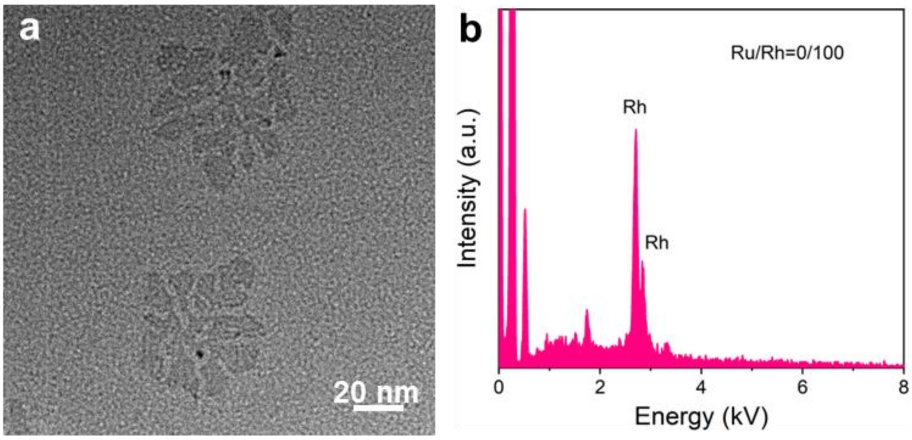

Figure S11. (a) TEM image and (b) HTEM-EDS spectrum of RhRu nanovesicles intermediates obtained at the reaction time of 50 min.

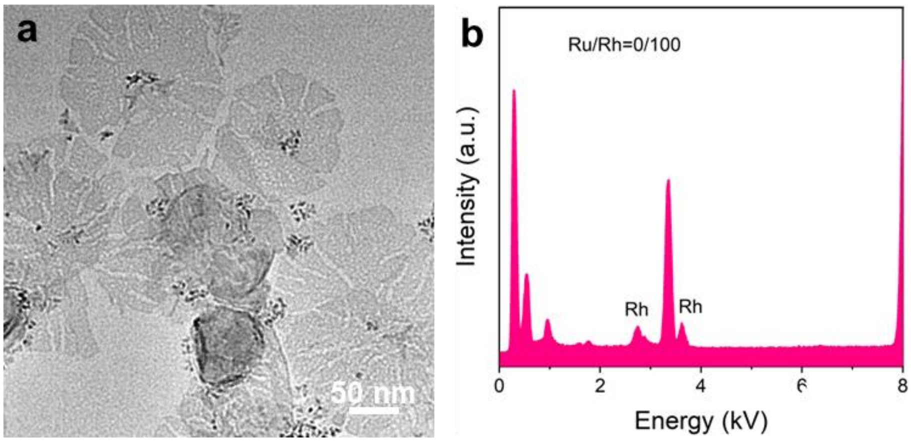

Figure S12. (a) TEM image and (b) HTEM-EDS spectrum of RhRu nanovesicles intermediates obtained at the reaction time of 60 min.

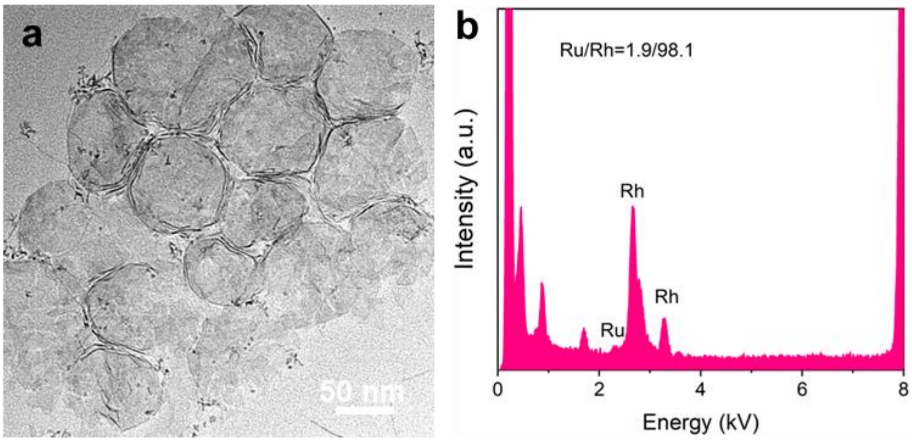

Figure S13. (a) TEM image and (b) HTEM-EDS spectrum of RhRu nanovesicles intermediates obtained at the reaction time of 75 min.

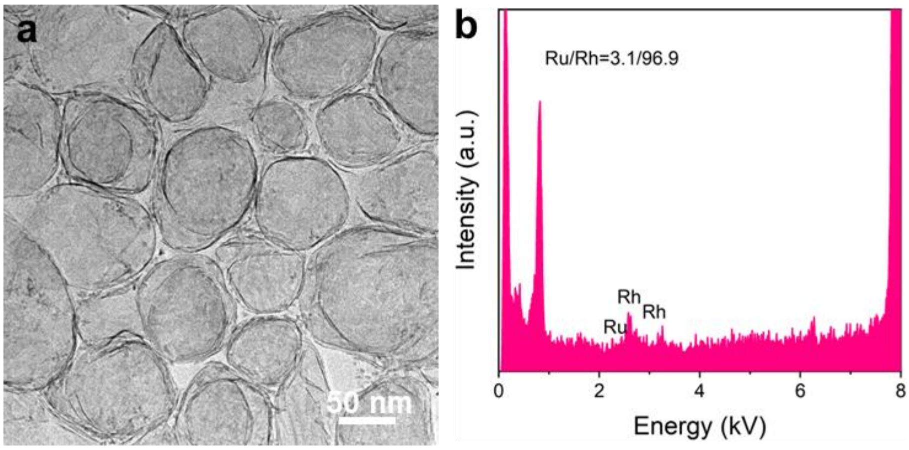

Figure S14. (a) TEM image and (b) HTEM-EDS spectrum of RhRu nanovesicles intermediates obtained at the reaction time of 180 min.

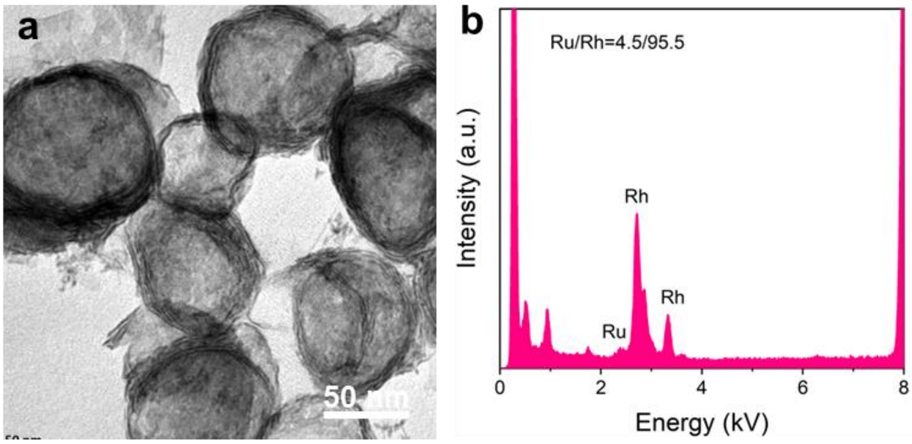

Figure S15. (a) TEM image and (b) HTEM-EDS spectrum of RhRu nanovesicles intermediates obtained at the reaction time of 300 min.

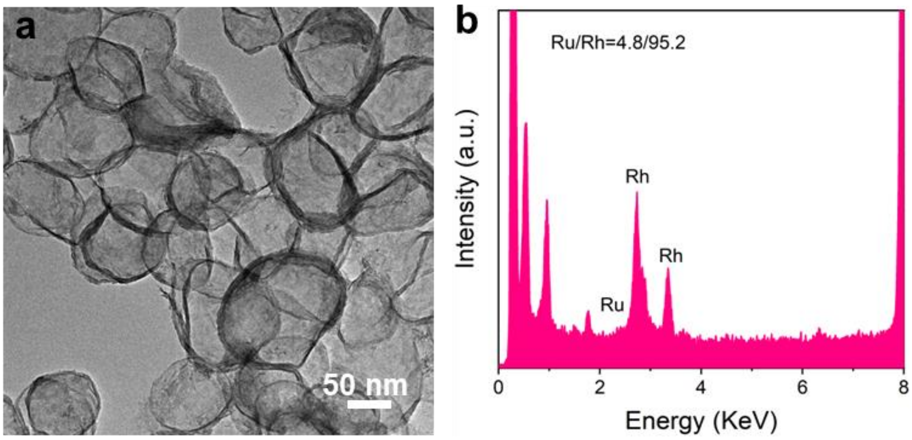

Figure S16. (a) TEM image and (b) HTEM-EDS spectrum of RhRu nanovesicles intermediates obtained at the reaction time of 480 min.

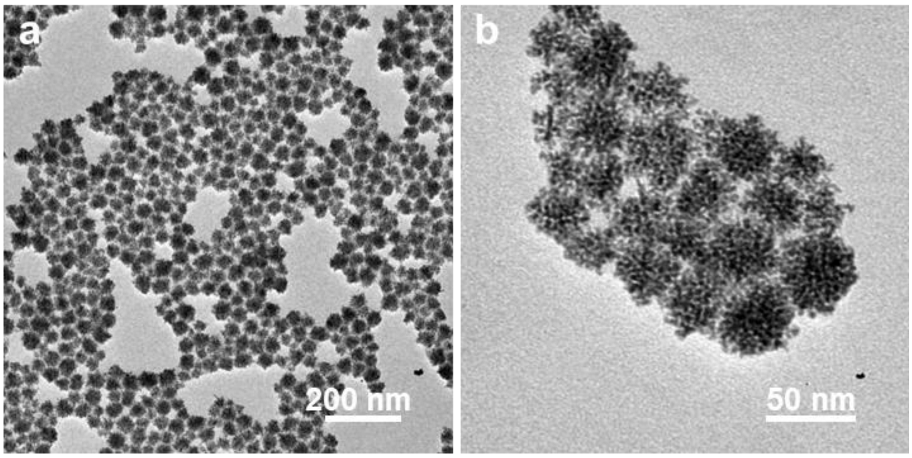

Figure S17. (a, b) TEM images of products with the same reaction conditions as those of RhRu nanovesicles except the use of 0 mg $Ru_3(CO)_{12}$.

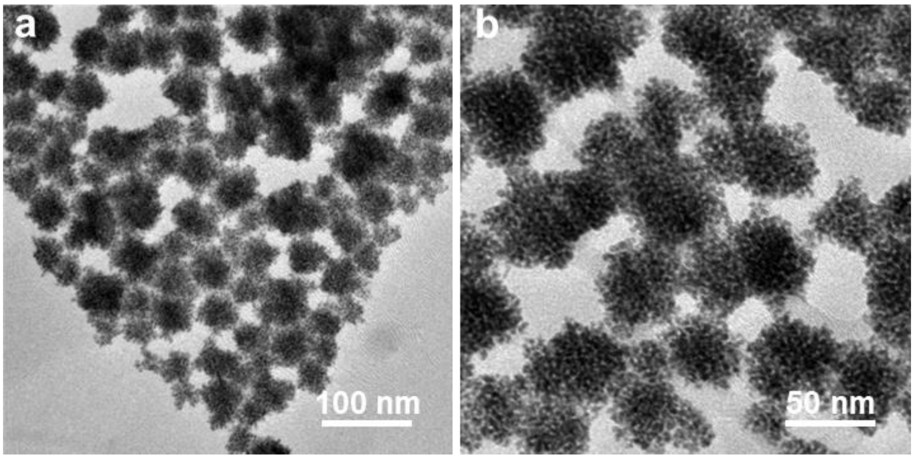

Figure S18. (a, b) TEM images of products with the same reaction conditions as those of RhRu nanovesicles except replacing $Ru_3(CO)_{12}$ with $Ru(acac)_3$.

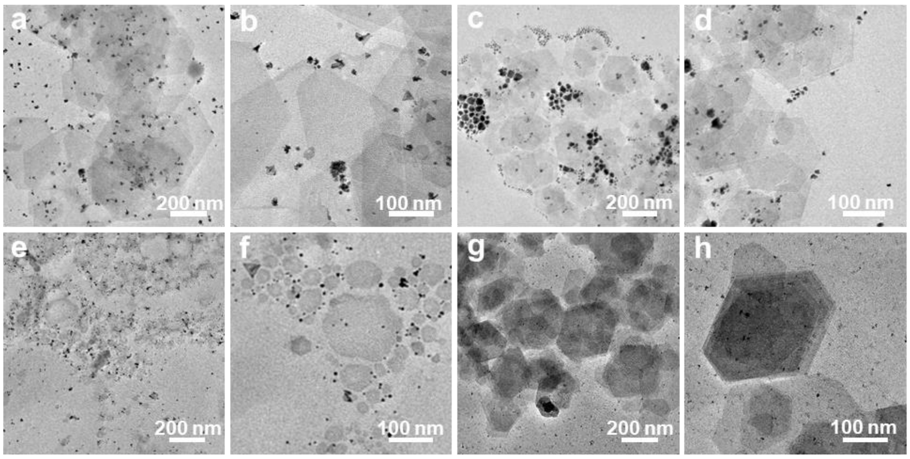

Figure S19. TEM images of products with the same reaction conditions as those of RhRu nanovesicles except replacing $Ru_3(CO)_{12}$ with (a, b) $Fe_2(CO)_9$, (c, d) $Co_2(CO)_8$, (e, f) $W(CO)_6$, and (g, h) $Mo(CO)_6$.

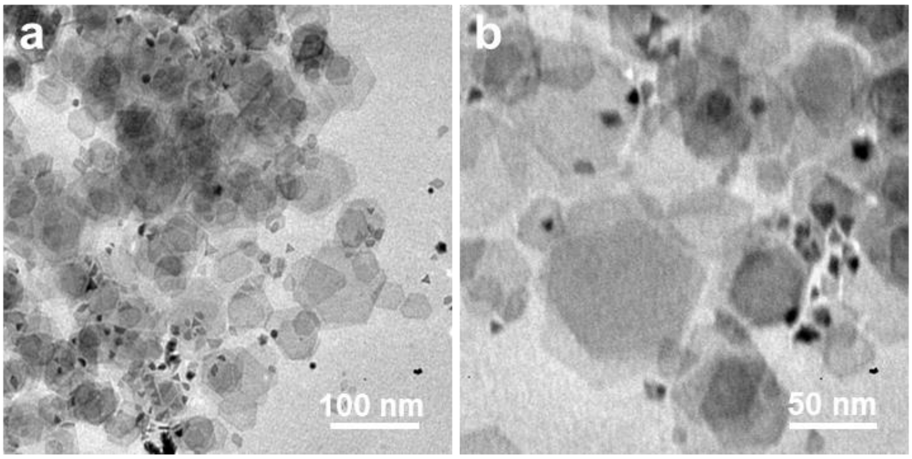

Figure S20. (a, b) TEM images of products synthesized by using $Rh(acac)_3$ as the metallic precursor, HCHO as the reductant, and diphenyl ether and oleylamine as solvent. The mixture was added into an autoclave and heated at 180 ℃ for 5 h.

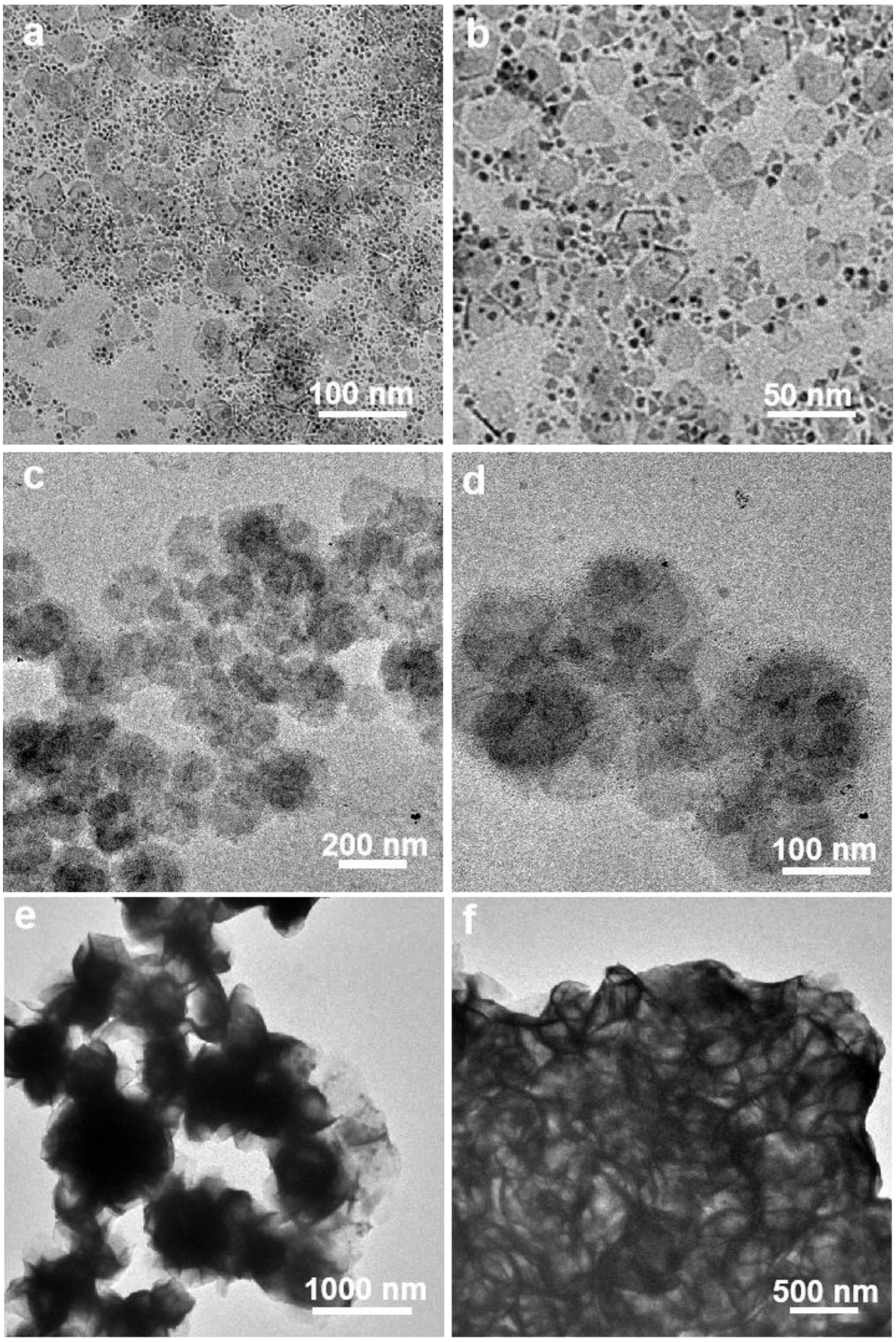

Figure S21. (a, b) TEM images of the product by removing CA. TEM images of the products by replacing CA with (c, d) ascorbic acid and (e, f) glucose.

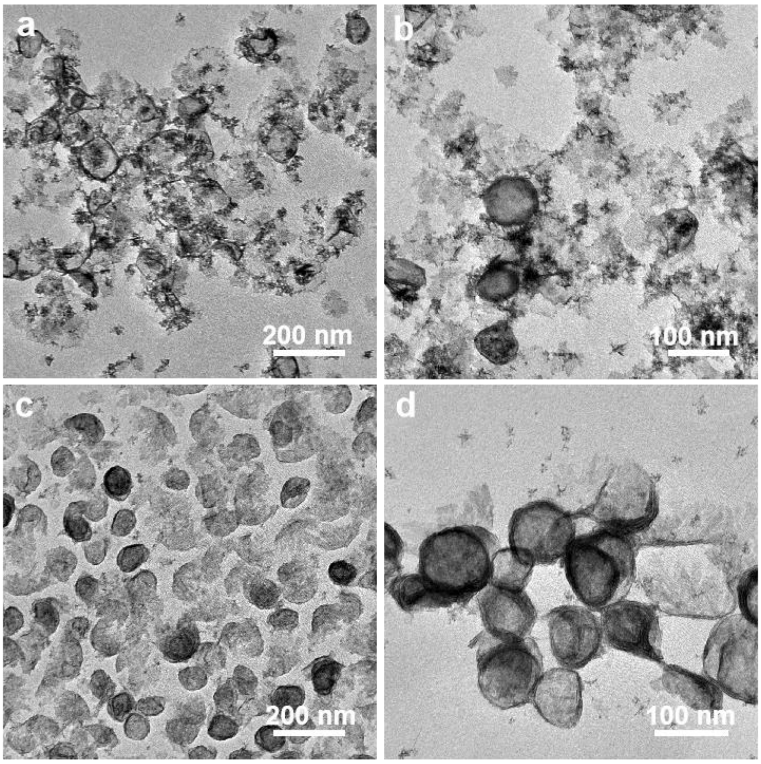

Figure S22. TEM images of the product synthesized with the reaction temperature of (a, b) 200 ℃, and (c, d) 120 ℃.

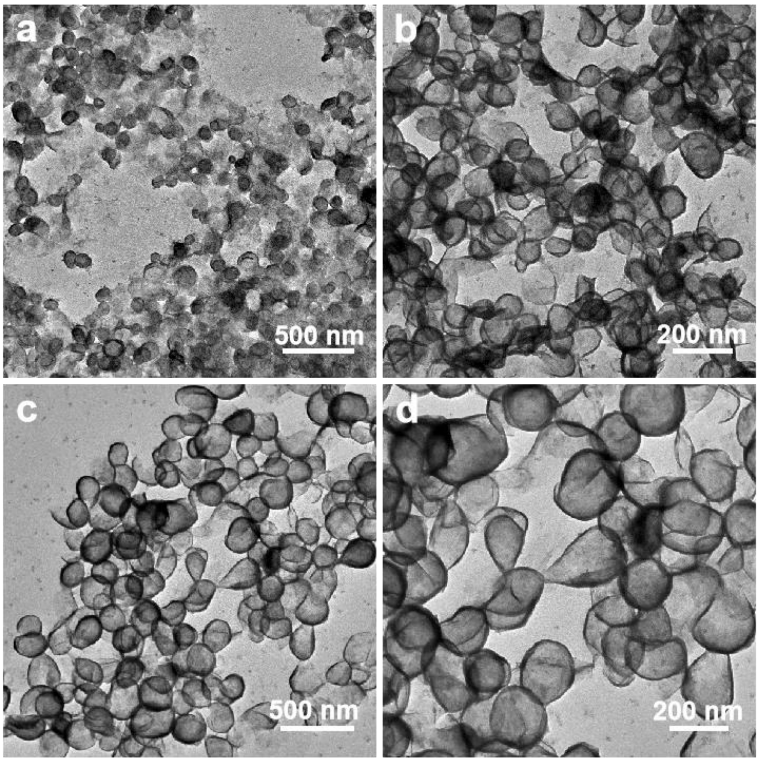

Figure S23. TEM images of the product synthesized by use of (a, b) 5 mg, and (c, d) 30 mg $Rh(acac)_3$.

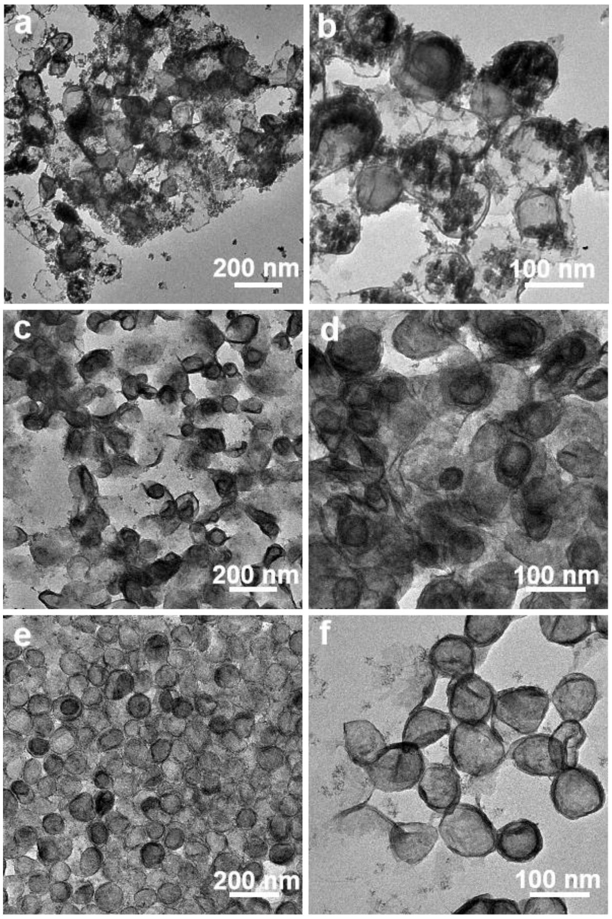

Figure S24. TEM images of the product synthesized with (a, b) 0 mg, (c, d) 16 mg, and (48 mg) KBr.

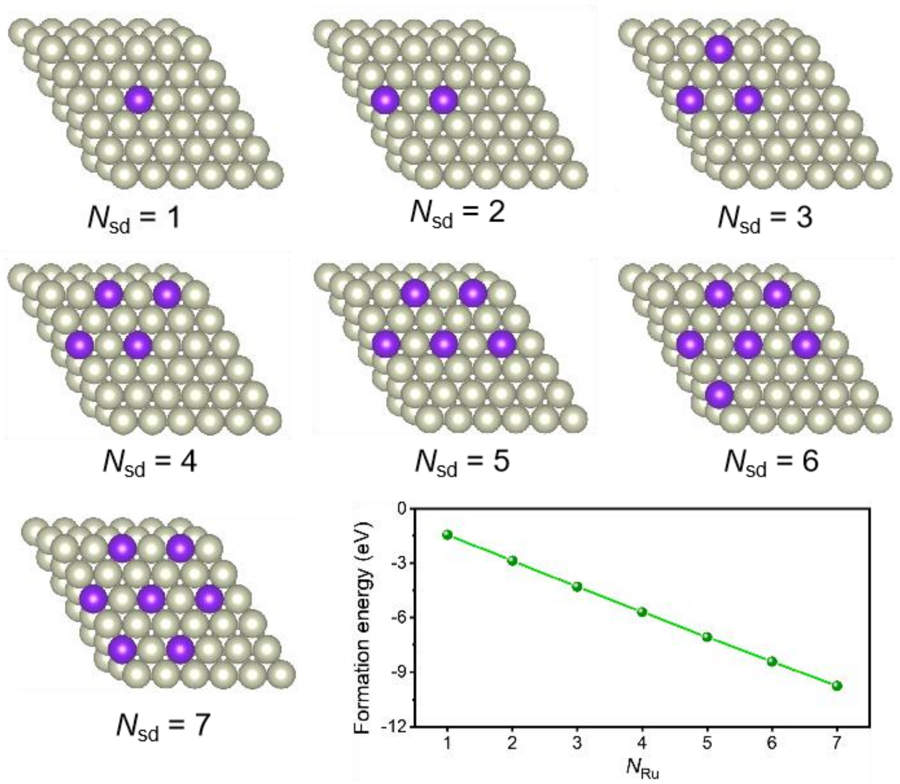

Figure S25. Atomic models of different configurations of Ru atoms in the alloy slab and corresponding the formation energy (E_f) of substitution defects in different configurations.

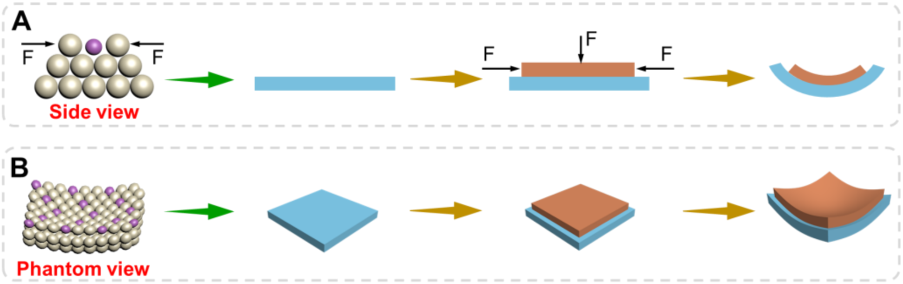

Figure S26. Simplified model diagram of curling process from (A) side and (B) phantom view.

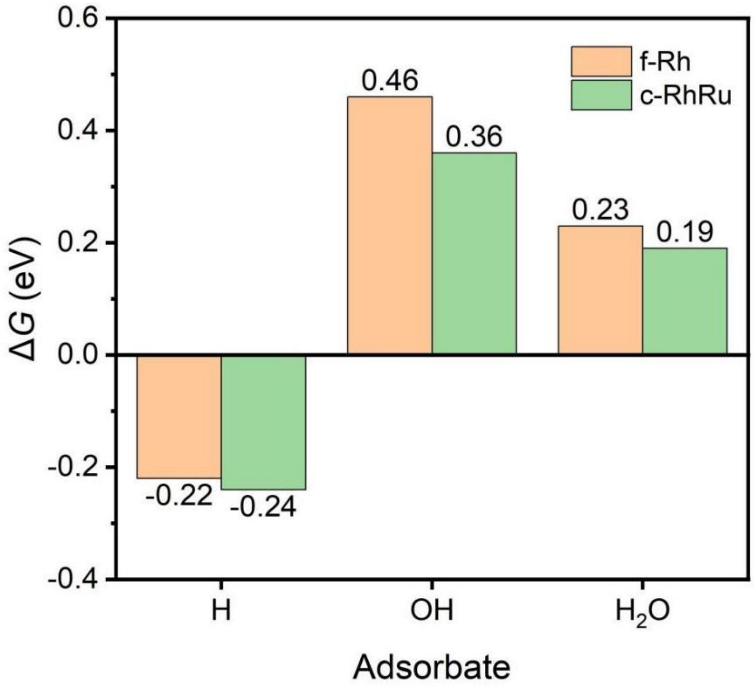

Figure S27. The Gibbs free energy (ΔG) changes before and after binding with H/OH/H_2O for f-Rh and c-RuRh.

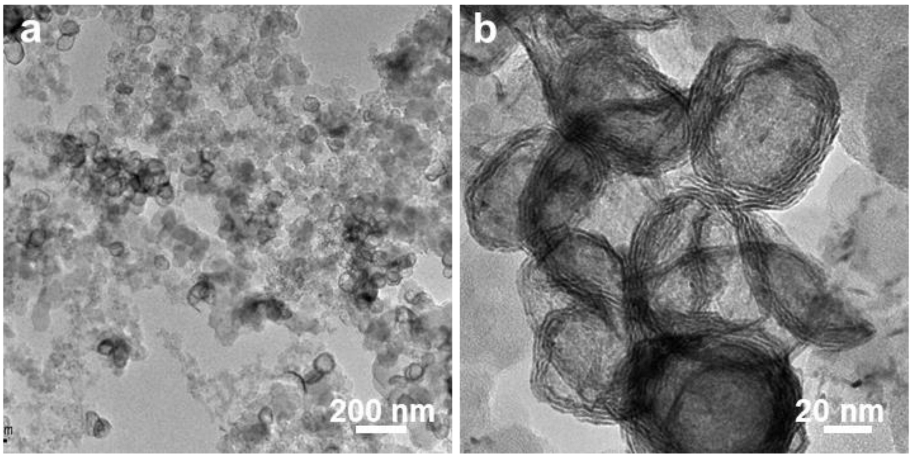

Figure S28. (a, b) TEM images of RhRu nanovesicles/C.

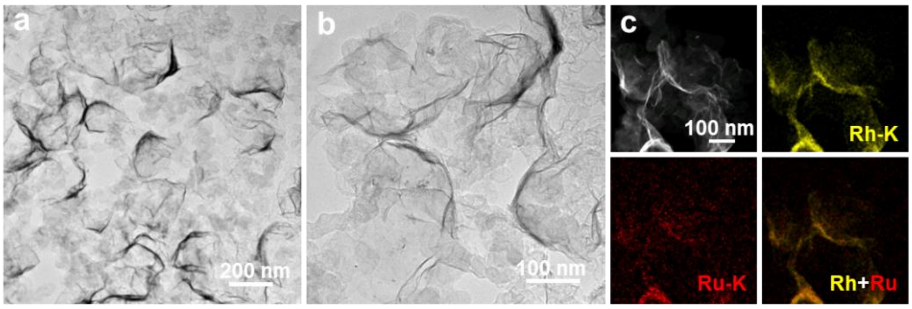

Figure S29. (a, b) TEM images, and (c) HAADF-STEM image with EDS elemental mappings of RhRu nanosheets/C.

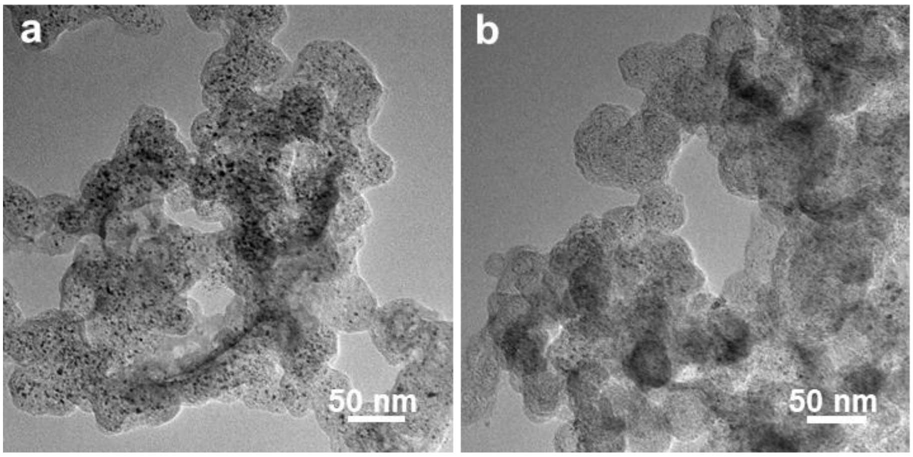

Figure S30. TEM images of (a) Pt/C and (b) Rh/C.

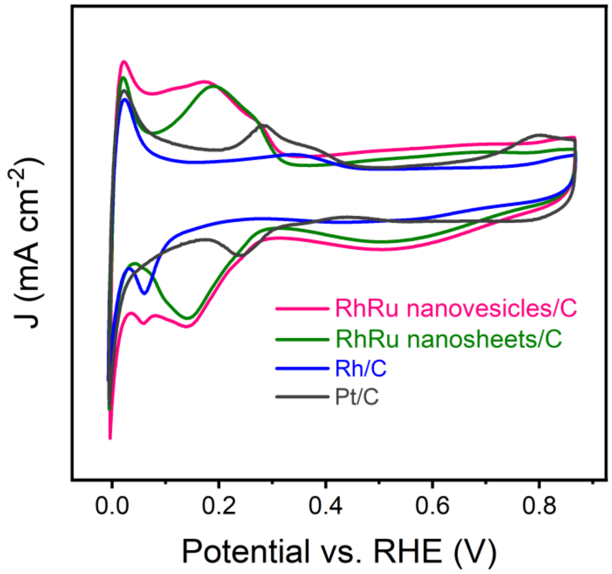

Figure S31. Polarization curves of RhRu nanovesicles/C, RhRu nanosheets/C, Rh/C, and Pt/C in N_2-saturated 0.1 M KOH at a scan rate of 50 mV s^{-1}.

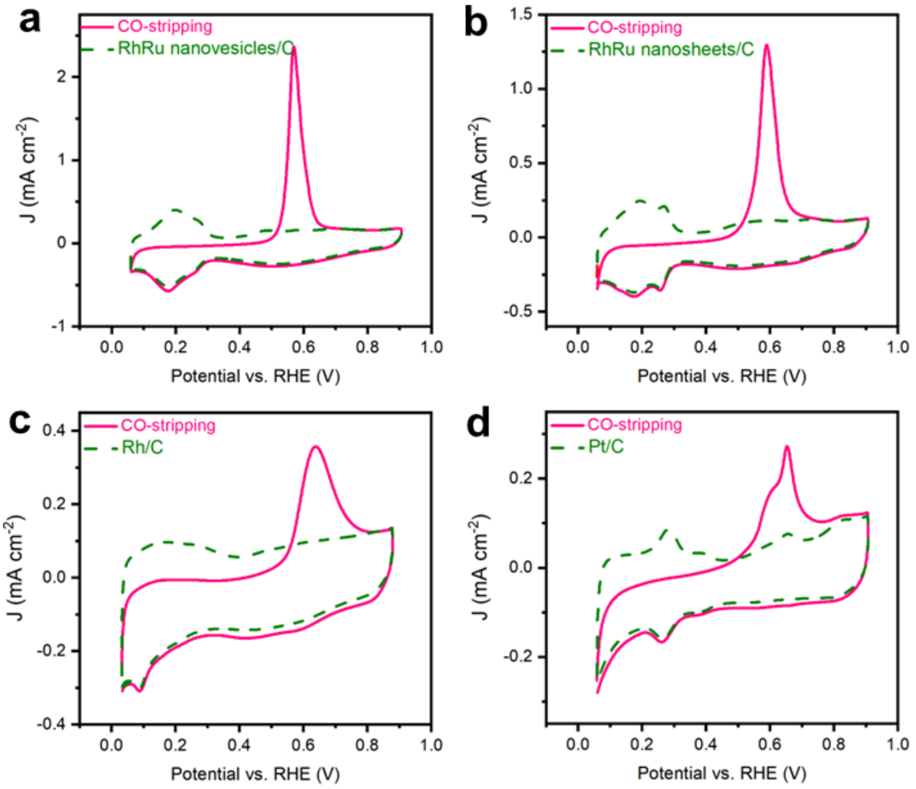

Figure S32. CO stripping voltammograms of (a) RhRu nanovesicles/C, (b) RhRu nanosheets/C, (c) Rh/C, and (d) Pt/C in 0.1 M KOH at a scan rate of 10 mV s^{-1}.

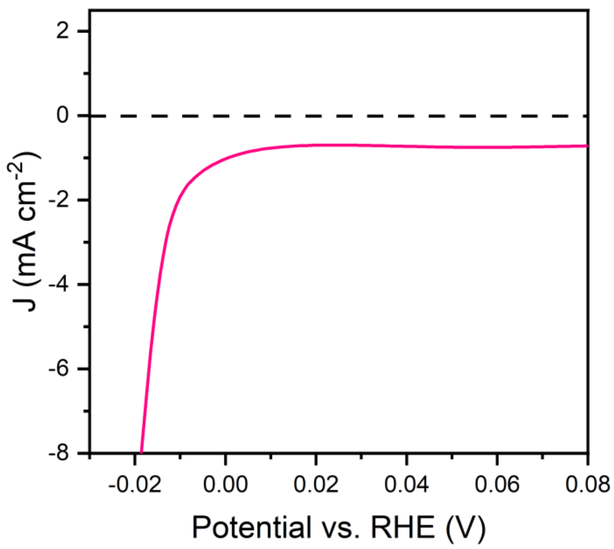

Figure S33. Polarization curve of RhRu nanovesicles/C in N_2-satuated 0.1 M KOH.

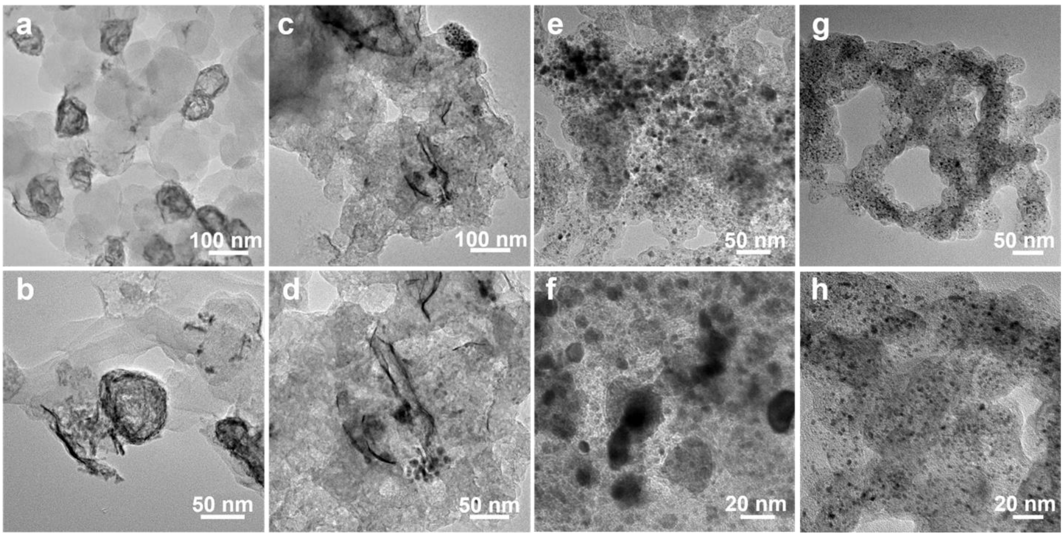

Figure S34. TEM images of (a, b) RhRu nanovesicles/C, (c, d) RhRu nanosheets/C, (e, f) Rh/C, and (g, h) Pt/C after 20000 s i-t tests.

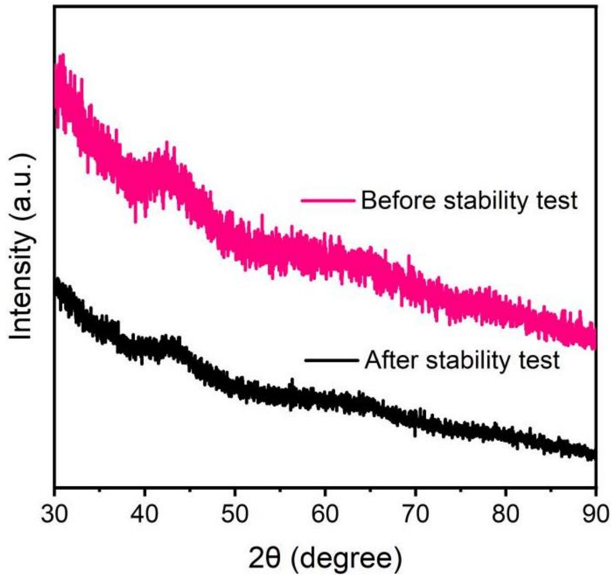

Figure S35. The XRD patterns of RhRu nanovesicles before and after HOR durability test.

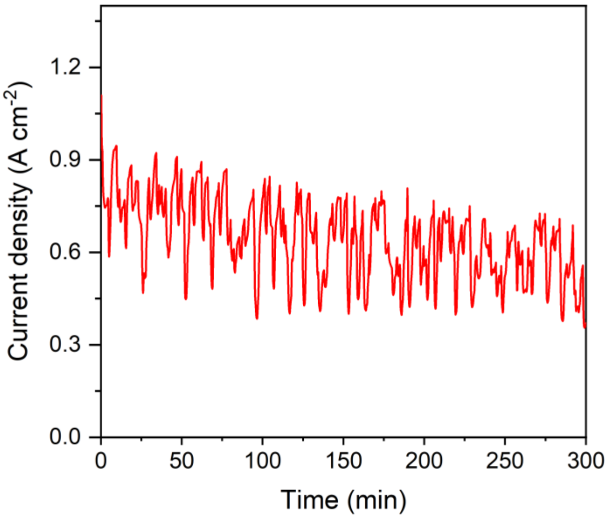

Figure S36. H_2-O_2 HEMFC current density degradation vs. time of RhRu nanovesicles/C at the voltage of 0.7 V (RhRu nanovesicles/C in 0.4 $mg_{(Rh+Ru)}$ cm^{-2} in anode and commercial Pt/C (0.4 mg_{Pt} cm^{-2}, 20 wt% Pt/C) in cathode).

Table S1. Structure parameters extracted from the Rh K-edge EXAFS and Ru K-edge EXAFS fitting ($S_0^2{}_{Rh}$=0.86, $S_0^2{}_{Ru}$=0.81)

**Samples**	**Path**	**CN**	**R(Å)**	**σ^2 (Å)**	**R-factor**
Rh foil	Rh-Rh	12	2.689±0.002	0.004±0.001	0.7%
Rh_2O_3	Rh-O	6	2.011±0.003	0.004±0.001	0.6%
RuRh-Rh	Rh-O<br>Rh-Ru	3.1±0.1<br>2.7±0.2	2.107±0.005<br>2.674±0.004	0.005±0.001<br>0.005±0.002	1.3%
Ru foil	Ru-Ru	12	2.677±0.004	0.004±0.001	0.6%
RuO_2	Ru-O	6	1.977±0.004	0.002±0.001	0.9%
RuRh-Ru	Ru-O<br>Ru-O<br>Ru-Rh	1.3±0.2<br>3.2±0.2<br>1.4±0.1	1.873±0.004<br>2.042±0.002<br>2.671±0.003	0.005±0.002<br>0.005±0.002<br>0.006±0.001	0.6%

S_0^2 is the amplitude reduction factor; CN is the coordination number; R is interatomic distance; σ^2 is Debye-Waller factor; R factor is used to value the goodness of the fitting.

Table S2. Comparison of mass activity of RhRu nanovesicles/C with other previous reported PGM catalysts for HOR in 0.1 M KOH.

**Catalyst**	**Mass activity (A mg_{PGM}^{-1})**	**Ref.**
**RhRu nanovesicles/C**	**7.52**	**This work**
RhMo NSs/C	6.96	*Nat. Commun.*, **14**, 1761 (2023).
Ru_1Pt_n-single atom	4.71	*ACS Appl. Mater. Interfaces* **14**, 27814-27822 (2022).
Mo-Pt/NC	4.55	*Adv. Energy Mater.* **12**, 2103336 (2022).
PtNi-S	4.08	*Nano Res.* **15**, 5865-5872 (2022).
Pt_2Ir DNWs/C	3.48	*J. Mater. Chem. A* **10**, 18972-18977 (2022).
Pt6NCs/C	3.66	*Nat. Commun.* **13**, 1596 (2022).
Rh_2Sb	3.25	*Adv. Mater.* **33**, 2105049 (2021).
$Ru_{0.96}Pt_{0.04}$ NTs	2.58	*ACS Catal.* **5**, 7015-7023 (2015).
PtRu NWs	2.2	*ACS Catal.* **6**, 3895-3908 (2016).
$Pt_{0.1}Ru_{0.9}$	1.9	*Nat. Chem.* **5**, 300-306 (2013).
$Pt_{0.25}Ru_{0.75}$/N-C	1.65	*Adv. Mater. Interfaces* **7**, 2000310 (2020).
O-PdFe@Pt/C	1.15	*J. Mater. Chem. A* **6**, 11346 (2018).
Pu@Pt NPs	1.03	*ACS Catal.* **5**, 6764 (2015).
BCC PdCu/C	0.77	*J. Am. Chem. Soc.* **140**, 16580-16588 (2018).
$Pt_{0.8}Ru_{0.2}$/C	0.696	*J. Phys. Chem. C* **119**, 13481-13487 (2015).
Pd_3Co@Pt/C	0.69	*Nano Energy* **74**, 104877 (2020).
RhSn/C	0.59	*J. Mater. Chem. A* **10**, 21856-21861 (2022).
Pt/Cu NWs	0.65	*J. Am. Chem. Soc.* **135**, 13473-13478 (2013).
Acid-PtNi/C	0.474	*J. Am. Chem. Soc.* **139**, 5156-5163 (2017).
PtRu/Mo_2C-TaC	0.403	*ACS Catal.* **11**, 932-947 (2021).
$Ru_{0.9}Pt_{0.1}$ NTs	0.24	*ACS Catal.***5**, 7015-7523 (2015).

Table S3. Comparison of peak power density of HEMFC (H_2-O_2) operated with PGM-anode catalysts.

Anode	Metal loading (mg_{PGM} cm^{-2})	Cathode ($mg\ cm^{-2}$)	PPD ($mW\ cm^{-2}$)	Ref.
**RhRu nanovesicles/C**	**0.4**	**0.4 (Pt/C)**	**1627**	**This work**
RhMo NSs	0.2	0.2 (Pt/C)	1520	*Nat. Commun.* **14**, 1761 (2023)
RuNi/NC	0.2	0.2 (Pt/C)	540	*Sci. Adv.* **8**, eabm3779 (2022)
Pd-CeO_2/OLC	0.15	0.4 (Pt/C)	1000	*ACS Catal.* **12**, 7014-7029 (2022).
Pd-CeO_2	0.15	0.4 (Pt/C)	900	
Pd–CeO_2/C	0.2	0.4 (Pt/C)	1400	*ACS Appl. Energy. Mater.* **2**, 4999-5008 (2019).
IrNi@PdIr/C	0.1	0.3 (Pt/C)	311	*Nanoscale* **10**, 4872-4881 (2018).
$Pd_{0.33}Ir_{0.67}$/C	0.2	0.3 (Pt/C)	514	*J. Mater. Chem. A* **7**, 3161-3169 (2019).
Ru/C	0.5	0.5 (Pt/C)	250	*J. Power Sources* **225**, 311-315 (2013).
Pd-CeO_2/C	0.42	0.58 (Pt/C)	1000	*J. Electrochem. Soc.* **165**, J3039-J3044 (2018).
CeO_x-Pd/C	0.38	0.7 (Pt/C)	1169	*Adv. Funct. Mater.* **30**, 2002087 (2020).
PtRu/N-C	0.7	0.4 (Pt/C)	831	*Adv. Mater. Interfaces* **7**, 2000310 (2020).
Ru_7Ni_3/C	0.2	0.4 (Pt/C)	2030	*Nat. Commun.* **11**, 5651 (2020).
PtRu/C	0.6	0.4 (Pt/C)	2550	*Energy Environ Sci.* **12**, 1575-1579 (2019).
Ru/meso C	0.1	0.45 (Pt/C)	1020	*J. Power Sources* **461**, 2002087 (2020).
RuNi/NC	1	0.2 (Pt/C)	540	*Sci. Adv.* **8**, eabm3779 (2022).

PtRu/C	0.4	0.4 (Pt/C)	1890	*J. Power Sources* **166**, F3305-F3310 (2019).
PtRu/C	0.6	Ag/C	1100	*Chem. Commun.* **53**, 11771-11773 (2017).
Pd/C-CeO_2	0.3	Ag/C	500	*Angew. Chem. Int. Ed.* **55**, 6004-6007 (2016).
Pd/Ni	0.3	Ag/C	400	*J. Power Sources* **304**, 332-339 (2016).
PtRu/C	0.4	$CoMn_2O_4$/C	1100	*ACS Energy Lett.* **4**, 1251-1257 (2019).
Pt/C	0.4	Mn-Co spinel/C	1100	*Nat. Commun.* **10**, 1506 (2019).
Pd/CeO_2-C	0.25	Ag-Co	1000	*ACS Appl. Energy Mater.* **3**, 10209-12214 (**2020**).
PtRu/C	0.4	Fe/N/C	400	*ACS Catal.* **7**, 6458-6492 (2017).
PdIrRu/C	0.2	Ag/C	820	*Chem. Commun.* **56**, 5669-5672 (2020).
PtRu/C	0.9	$Fe_{0.5}$-NH_3	1040	*J. Electrochem. Soc.* **167**, 134505 (2020).
